# Multi-cohort validation based on a novel prognostic signature of anoikis for predicting prognosis and immunotherapy response of esophageal squamous cell carcinoma

**DOI:** 10.3389/fonc.2025.1530035

**Published:** 2025-03-17

**Authors:** Zhongquan Yi, Xia Li, Yangyang Li, Rui Wang, Weisong Zhang, Hao Wang, Yanan Ji, Jing Zhao, JianXiang Song

**Affiliations:** ^1^ Department of Central Laboratory, Affiliated Hospital 6 of Nantong University, Yancheng Third People's Hospital, Yancheng, China; ^2^ Department of General Medicine, Affiliated Hospital 6 of Nantong University, Yancheng Third People's Hospital, Yancheng, China; ^3^ Department of Cardiothoracic Surgery, Affiliated Hospital 6 of Nantong University, Yancheng Third People’s Hospital, Yancheng, China

**Keywords:** esophageal squamous cell carcinoma, anoikis, prognostic signature, immunotherapy, tumor immune microenvironment

## Abstract

Immunotherapy is recognized as an effective and promising treatment modality that offers a new approach to cancer treatment. However, identifying responsive patients remains challenging. Anoikis, a distinct form of programmed cell death, plays a crucial role in cancer progression and metastasis. Thus, we aimed to investigate prognostic biomarkers based on anoikis and their role in guiding immunotherapy decisions for esophageal squamous cell carcinoma (ESCC). By consensus clustering, the GSE53624 cohort of ESCC patients was divided into two subgroups based on prognostic anoikis-related genes (ARGs), with significant differences in survival outcomes between the two subgroups. Subsequently, we constructed an ARGs signature with four genes, and its reliability and accuracy were validated both internally and externally. Additional, different risk groups showed notable variances in terms of immunotherapy response, tumor infiltration, functional enrichment, immune function, and tumor mutation burden. Notably, the effectiveness of the signature in predicting immunotherapy response was confirmed across multiple cohorts, including GSE53624, GSE53625, TCGA-ESCC, and IMvigor210, highlighting its potential utility in predicting immunotherapy response. In conclusion, the ARGs signature has the potential to serve as an innovative and dependable prognostic biomarker for ESCC, facilitating personalized treatment strategies in this field, and may represent a valuable new tool for guiding ESCC immunotherapy decision-making.

## 1 Introduction

Esophageal cancer (EC) is an upper gastrointestinal tract malignancy, with more than 470,000 new cases diagnosed each year, which accounts for over 3% of global cancer incidence (1–4). Esophageal squamous cell carcinoma (ESCC) is the most common form of EC, making up approximately 90% of cases (5, 6). Since ESCC is typically diagnosed at an advanced stage or metastatic, there are few treatment options available and the 5-year mortality rate is high (4, 7–10). Recently, immunotherapy has advanced rapidly (11–14), demonstrating promising immunotherapy outcomes in patients with ESCC (15–19). However, owing to the substantial heterogeneity of ESCC arising from variations in both tumor cells and the tumor environment, the clinical response rate remains low, and only a small portion of cases benefit from treatment (20, 21). Therefore, identifying ESCC patients who probably benefit from immunotherapy is critical to improving clinical outcomes.

The tumor immune microenvironment (TME) plays a critical role in the development, progression, metastasis, and response to therapies of tumors (22–27). The TME is a dynamic and complex multicellular context that emerges from the interaction between tumor cells and the stroma (25, 28, 29). Recently, Zheng et al. (30) observed that the TME in ESCC is abundant in immune-suppressive cell populations, encompassing regulatory T cells (Tregs), exhausted CD4 T, CD8 T, and NK cells, M2 macrophages, and tolerogenic dendritic cells. Among them, Tregs, which are characterized by the expression of Foxp3 and CD25, exert influences on various aspects of the anti-tumor immune response via their immunosuppressive properties (25, 31). Additionally, several studies have described that cancer-associated fibroblasts play a pivotal role in the in the formation of an immunosuppressive TME in ESCC (32–34). The increasing comprehension of the TME in ESCC patients will facilitate the understanding of the immune status of ESCC, which holds crucial practical implications for evaluating whether patients are responding to immunotherapy and for developing novel immunotherapy strategies (30, 35, 36).

Anoikis, a distinct form of programmed cell death, is triggered when cells detach from the extracellular matrix or surrounding cells, which effectively removes displaced cells and prevents detached cells from attaching incorrectly (37–39). In order for cancer cells to metastasize and invade, various pathways must be developed for cancer cells to develop inactivation resistance, evade cell death, and establish metastatic lesions (39, 40). In recent research, it has been found that prognostic signatures utilizing anoikis-related genes (ARGs) have significant value in predicting the prognosis of cancer patients and their response to immunotherapy (41–46). For example, Lei Yang and Feng Xu built an ARGs signature and found it to be a dependable indicator of prognosis and treatment response in patients with colorectal cancer (45). In glioblastoma, Sun et al. (46) constructed an ARGs signature and evaluated its value in survival prediction, tumor microenvironment (TME), and immunotherapy responses. Although the study by zhang et al. (47) provided insights into the role of ARGs in ESCC, there are still certain limitations that need to be addressed. Specifically, the potential of ARGs signature in predicting the prognosis in ESCC has been exclusively examined within the TCGA-ESCC cohort, lacking validation in other external cohorts. Furthermore, the capacity of ARG signature alone to predict immunotherapy response remains unexplored. Therefore, further investigation is necessary to elucidate the impact of ARGs signature on ESCC.

In this study, we utilized multiple cohorts from RNA transcriptome (including GSE53624, GSE53625, TCGA-ESCC, and IMvigor210 cohorts) and single-cell RNA sequence database (GSE188900) for analysis. Subsequently, a range of algorithms were utilized to construct an ARGs prognostic signature. This signature was used in multiple cohorts to predict ESCC prognosis and assess the efficacy of immunotherapy response. These analyses elucidate the role of the ARGs signature in ESCC and offer novel information for personalized cancer immunotherapy.

## 2 Materials and methods

### 2.1 Data processing

**Figure 1:**
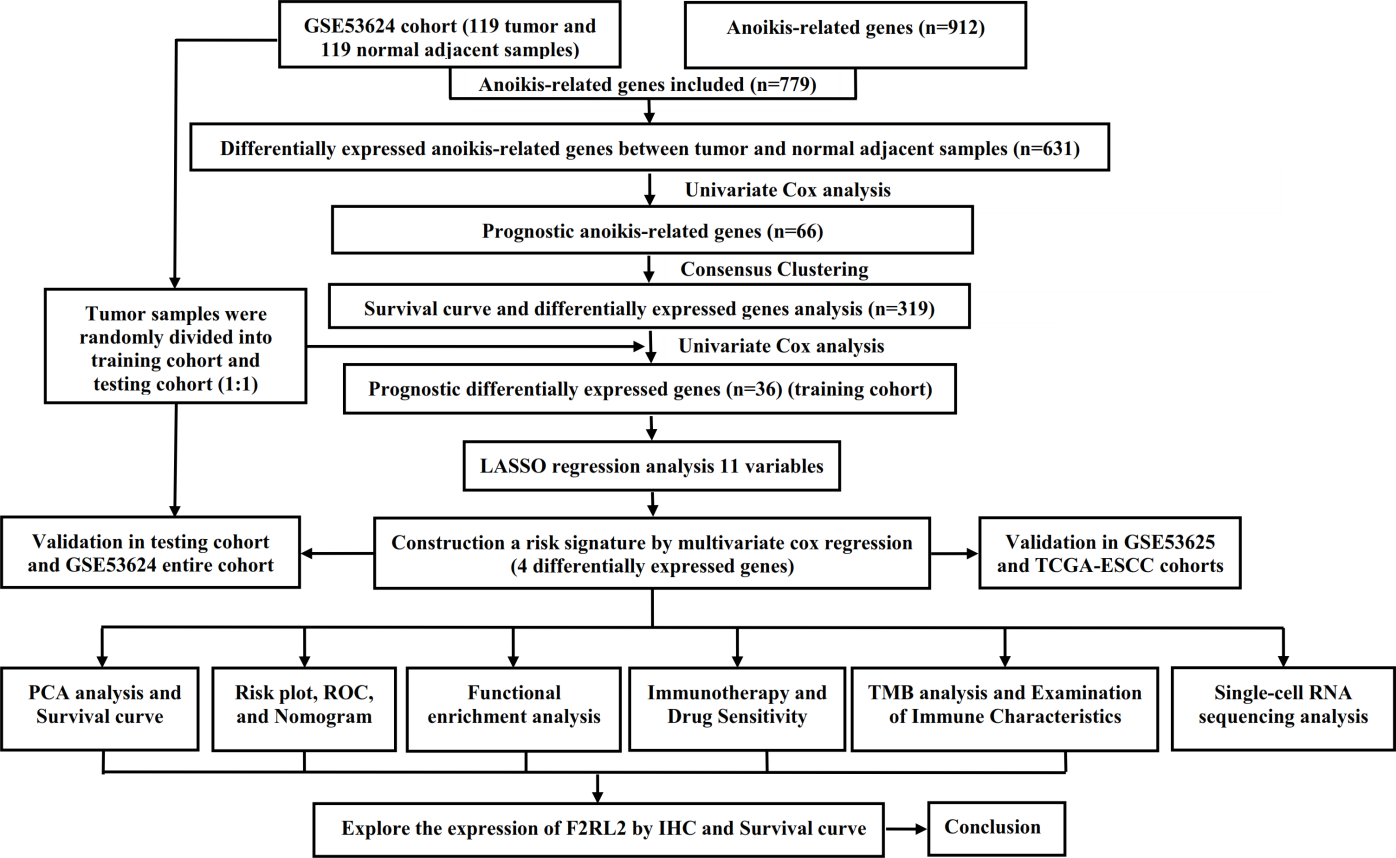
The flow chart of research design. ESCC, esophageal squamous cell carcinoma; PCA, principal component analysis; ROC, receiver operating characteristic; TMB, tumor mutational burden; IHC, Immunohistochemistry.

The general study process is detailed in **Figure 1**. From the Gene Expression Omnibus (GEO) database, we obtained the transcriptome data and clinical characteristics. The GSE53624 cohort encompassed 119 tumor samples and 119 normal adjacent samples. The tumor samples from the GSE53624 cohort was split into training (60 samples) and testing cohorts (59 samples) using the R package "caret" in a random manner. For external validation of prognostic signature, transcriptome data and associated clinical characteristics were extracted from GEO (GSE53625 cohort, n = 179) and TCGA (TCGA-ESCC cohort, n = 93) cohorts. In addition, we obtained IMvigor210 database through R package IMvigor210CoreBiologies (28) and selected samples with complete treatment response information (n = 298). A list of ARGs was obtained from previously published literature (48), and 779 ARGs were included for analysis after excluding genes absent in the GSE53624 cohort.

ESCC single-cell data were obtained from the GSE188900 dataset (sample size: n = 4). The Seurat v4.1 package was used to perform a standard pre-processing workflow for single-cell sequencing. Low-quality cells (mitochondrial genes > 20 %, gene numbers < 200, and gene numbers > 6000) were excluded from the subsequent analysis. Harmony (version 1.0) was employed to correct batch effects in the dataset comprising four samples and to integrate the merged objects. The t-distributed stochastic neighbor embedding (t-SNE) was utilized to visualize cell clusters.

### 2.2 Development and validation of the signature

In the GSE53624 cohort, we initially conducted a comparison of the expression levels of 779 ARGs between tumor and normal adjacent esophageal tissues. Subsequently, we conducted univariate cox analysis of differentially expressed ARGs to identify those associated with prognosis. Based on these prognostic ARGs, consensus clustering was conducted by employing the "ConsensusCluster Plus" R package with 1000 repetitions, 80% re-sampling, clusterAlg = "km", and distance = "euclidean". Then, a combination of the consensus score matrix, the CDF curve, and the PAC score was employed to determine the optimal number of clusters. The differentially expressed genes (DEGs) between subgroups were detected using the "limma" package, with criteria of |logFC| > 1 and FDR < 0.05. An ARGs signature was established using the GSE53624 training cohort and validated across multiple cohorts including the GSE53624 testing cohort, the GSE53624 entire cohort, the GSE53625 cohort, and TCGA-ESCC cohort. Prognostic DEGs were identified through univariate Cox regression in the GSE53624 training cohort followed by LASSO regression. The most effective prognostic signature was constructed using stepwise regression methods via multiple cox regression analyses. Risk score = ∑i=EXP (i) × Coef (i). Based on the medium risk score, each ESCC patient was classified as high- or low-risk accordingly. We utilized Kaplan-Meier analysis to assess the overall survival (OS) differences between subgroups in order to validate and evaluate performance. We used the ROC curve to assess their predictive ability (49). In addition, principal component analysis (PCA) was used to evaluate the grouping effect of ARGs signature (50). Taking into account various ESCC patients' clinical feature, Cox regression analyses were performed to investigate the potential of the ARGs signature as an independent risk factor.

### 2.3 Functional enrichment analysis

Utilizing the "limma" package, DEGs between risk groups were identified (|logFC| > 0.585 and FDR < 0.05) (51). Relevant analyses were implemented through "clusterProfiler" R package (52) to examine the potential function of DEGs, including GO and KEGG analyses. Additionally, to evaluate potential pathways for signaling and biological functional alterations between groups, Gene Set Enrichment Analysis (GSEA) were applied by utilizing KEGG and Hallmark gene sets (*p* < 0.05).

### 2.4 Analysis of immune cell infiltration, tumor mutational burden, exclusion, and drug sensitivity analysis

In this study, we utilized the ESTIMATE method to calculate the stromal, estimate, and immune scores for each ESCC sample, as well as the tumor purity score. Additionally, we evaluated the abundance of 22 immune cells infiltration between different risk groups was assessed through CIBERSORT algorithm (53). Furthermore, we utilized single sample gene set enrichment analysis (ssGSEA) algorithms to measure the infiltration of 22 immune cells and overall immune function. Correlations between risk score and 22 immune cells infiltration were assessed using Pearson correlation coefficient.

Utilizing the "maftools" package, we conducted an analysis of TMB data obtained from TCGA database (54). Furthermore, TIDE scores for each patient were acquired through the online website (55). Additionally, we used the R package "oncoPredict" to predict the IC50 value of potential therapeutic drugs for ESCC between risk groups (*p* < 0.05) (56).

### 2.5 Immunohistochemistry

We obtained a tissue microarray containing 80 pairs of ESCC tissues and their corresponding adjacent normal esophageal tissues from Changsha Xiangya Biotechnology Co., Ltd. After dewaxing, antigen repair, and blocking endogenous peroxidase, the microarray was left to incubate overnight at 4°C with anti-F2RL2 primary antibody (bs-9510P, Bioss, China). Subsequently, secondary antibody incubation and visualization of immunoreactivity with DAB (G1211, Servicebio, China) were performed. In order to ensure an impartial evaluation, two pathologists who were unaware of the clinical information independently assessed the IHC results and resolved any discrepancies through discussion. The H-score method was used to evaluate staining intensity and extent by taking into account both intensity (ranging from 0 to 3+) and the percentage of tumor cells that were positively stained (ranging from 0% to 100%). The level of F2RL2 expression was equal to the product of these two estimates.

### 2.6 Statistical analysis

To conduct the statistical analyses, GraphPad and R 4.3.1 were used. According to the specific circumstances, we employed either the student t-test or Wilcoxon rank sum test for intergroup comparisons. Pearson correlation coefficient was utilized to assess correlation between variables. Chi-square test was implemented to evaluate whether there are differences in clinical characteristics. All statistical tests were considered significant at *p* < 0.05.

## 3 Results

### 3.1 Identification and construction of ARGs signature

**Figure 2:**
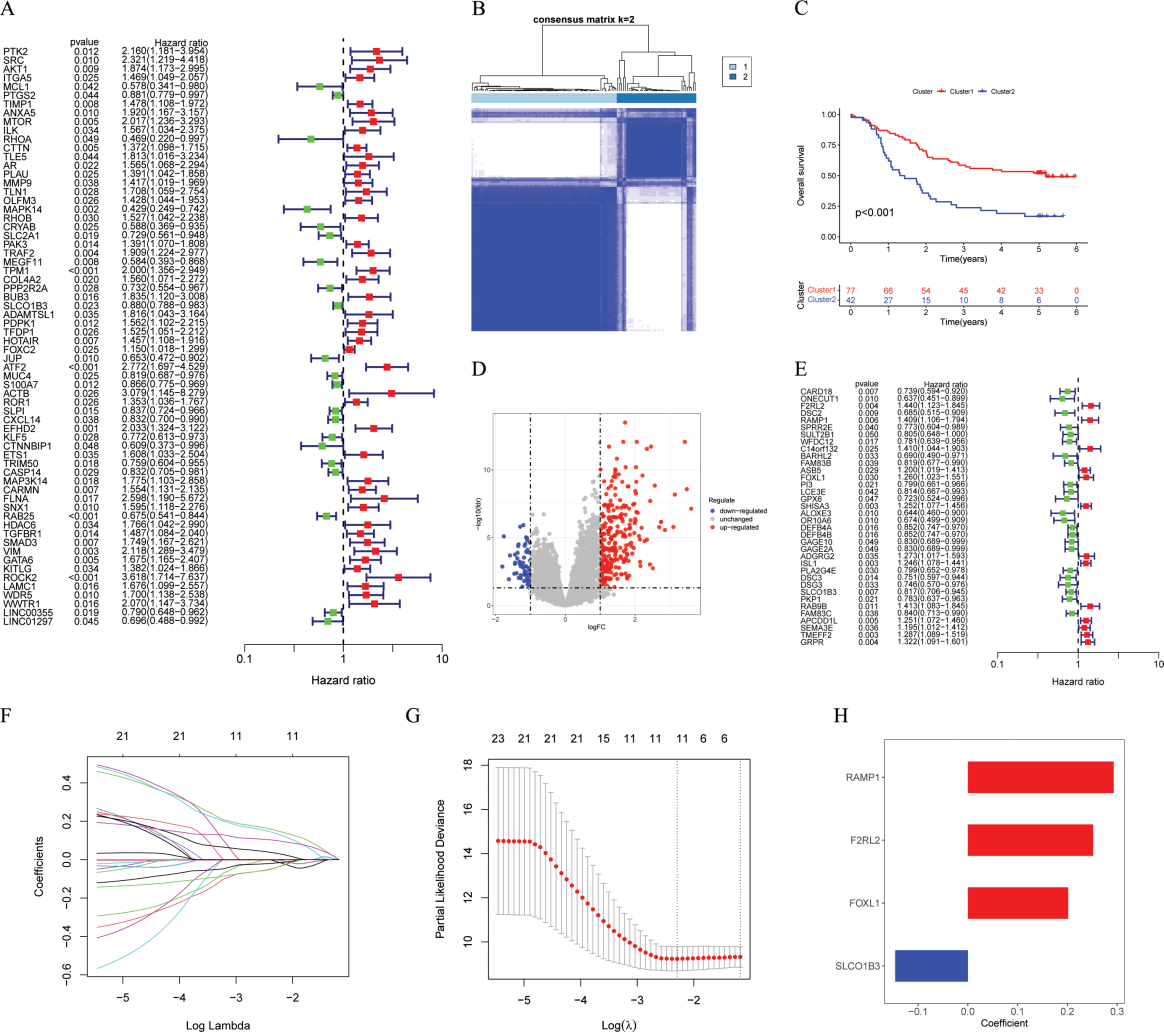
Identification and construction of the ARGs signature. **(A)** Univariate Cox analysis to determine potential prognostic ARGs (*p* < 0.05). **(B)** The consensus score matrix of GSE53624 cohort (n = 119) when k = 2. **(C)** The Kaplan-Meier survival curve showing different overall survival between the two clusters (*p* < 0.001), with Cluster 1 showing better outcomes. **(D)** The volcano plot showing DEGs between the two clusters with criteria of |logFC| > 1 and FDR < 0.05. **(E)** Univariate Cox analysis to determine potential prognostic DEGs (*p* < 0.05). **(F, G)** The coefficient profile of prognostic DEGs by Lasso regression analysis. The optimal λ was obtained when the partial likelihood deviance reached the minimum value. **(H)** Multivariate Cox coefficients for 4 DEGs (RAMP1, F2RL2, FOXL1, and SLCO1B3) in the ARGs signature. ARGs, anoikis-related genes; DEGs, different expression genes.

After conducting differential analysis between tumor and normal adjacent samples, 631 differentially expressed ARGs were identified (**Supplementary Table S1**). Through univariate Cox regression analyses, 66 prognostic ARGs were identified (**Figure 2A**). Subsequently, we conducted a consensus cluster analysis (k = 2-6) and found that the optimal number was obtained when k = 2 (**Figure 2B**). KM analysis showed significant differences in prognosis between two clusters (**Figure 2C**). Next, we conducted differential analysis between two clusters and identified 319 DEGs (**Figure 2D**; **Supplementary Table S2**). Subsequently, 119 ESCC patients were divided into two cohorts: training (n = 60) and testing (n = 59), maintaining an approximate 1:1 ratio. **Table 1** displays the baseline characteristics of both the training and testing cohorts, showing no significant disparities. Through univariate Cox regression analysis, 36 prognostic DEGs were selected (*p* < 0.05) in the training cohort (**Figure 2E**). Subsequent LASSO regression analysis identified a minimum lambda value of 11 (**Figures 2F, G**). After multivariate Cox regression screening, an ARGs signature was developed based on four DEGs (**Figure 2H**) (RAMP1, F2RL2, FOXL1, and SLCO1B3). Following formula: Risk score = RAMP1 * 0.29303 + F2RL2 * 0.25188 + FOXL1 * 0.20157 + SLCO1B3 * (-0.14595), each ESCC patient's risk score was computed.

**Table 1:** Comparisons of patient characteristics between training and testing cohorts.

Characteristics	Total cohort (*n* = 119)	Training cohort (*n* = 60)	Testing cohort (*n* = 59)	*P-*value
Age				
≤60	69 (57.98%)	39 (65%)	30 (50.85%)	0.118
> 60	50 (42.02%)	21 (35%)	29 (49.15%)	
Gender				
Male	98 (82.35%)	48 (80%)	50 (84.75%)	0.497
Female	21 (17.65%)	12 (20%)	9 (15.25%)	
T stage				
T1	8 (6.72%)	3 (5%)	5 (8.48%)	0.214
T2	20 (16.81%)	8 (13.33%)	12 (20.34%)	
T3	62 (52.1%)	37 (61.37%)	25 (42.37%)	
T4	29 (24.37%)	12 (20%)	17 (28.81%)	
N stage				
N0	54 (45.38%)	29 (48.34%)	25 (42.37%)	0.806
N1	42 (35.29%)	21 (35%)	21 (35.59%)	
N2	13 (10.92%)	5 (8.33%)	8 (13.56%)	
N3	10 (8.41%)	5 (8.33%)	5 (8.48%)	
TNM stage				
I	6 (5.04%)	3 (5%)	3 (5.08%)	0.808
II	47 (39.5%)	22 (36.67%)	25 (42.37%)	
III	66 (55.46%)	35 (58.33%)	31 (52.55%)	

### 3.2 Prognosis prediction of ESCC patients utilizing the ARGs signature

**Figure 3:**
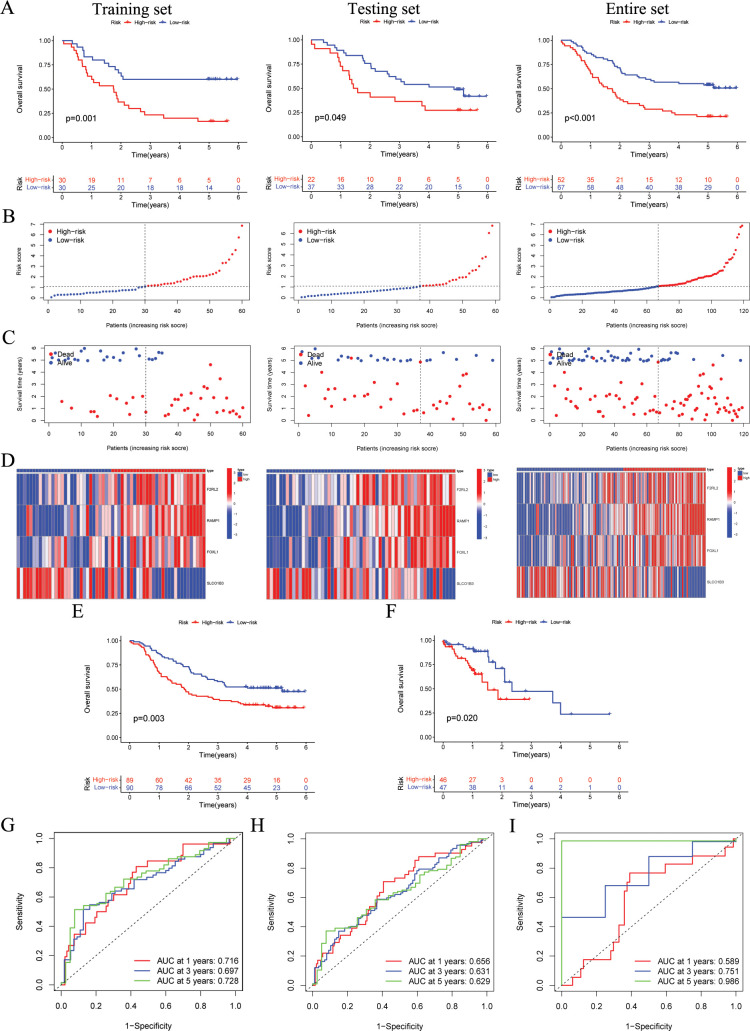
The validation of the ARGs signature in both internal and external cohorts. **(A)** OS of patients in different risk groups in the GSE53624 training (n = 60, *p* = 0.001), testing (n = 59, *p* = 0.049), and entire (n = 119, *p* < 0.001) cohorts, with low ARGs group showing better outcomes. **(B, C)** The distribution of risk scores and OS status for each patients in the GSE53624 training, testing, and entire cohorts. **(D)** Heatmap showing the expression of the four modeling genes in the GSE53624 training, testing, and entire cohorts. **(E)** OS of patients in different risk groups in the GSE53625 cohort (n = 179, *p* = 0.003), with low ARGs group showing better outcomes. **(F)** OS of patients in different risk groups in the TCGA-ESCC cohort (n = 93, *p* = 0.020), with low ARGs group showing better outcomes. **(G-I)** ROC curves for predicting 1-, 3-, and 5-year OS in the GSE53624, GSE53625, and TCGA-ESCC cohorts. ARGs, anoikis-related genes; OS, overall survival; ROC, Receiver operating characteristic; ESCC, esophageal squamous cell carcinoma.

In accordance with the median score of training cohort, each ESCC patient was classified as either the high-risk or low-risk within their respective cohorts for GSE53624 training, GSE53624 testing, and GSE53624 entire. The heatmap of four modeling genes expression and the distribution of OS status and risk score were presented in **Figures 3B–D**. After conducting KM analysis, it was noted that the low-risk ESCC group exhibited better OS in comparison to the high-risk ESCC group across all cohorts (*p* < 0.05) (**Figure 3A**). Following internal validation, GSE53625 and TCAG-ESCC were selected as external cohorts to reconfirm the predictive effects of the signature. According to the results, both external validation and internal validation cohorts exhibit good cross-validation effects (**Figures 3E, F**). ROC analysis revealed that risk scores exhibited high levels of specificity and sensitivity in both the internal and external cohorts (**Figures 3G–I**).

### 3.3 Independent prognosis of ARGs signature

**Figure 4:**
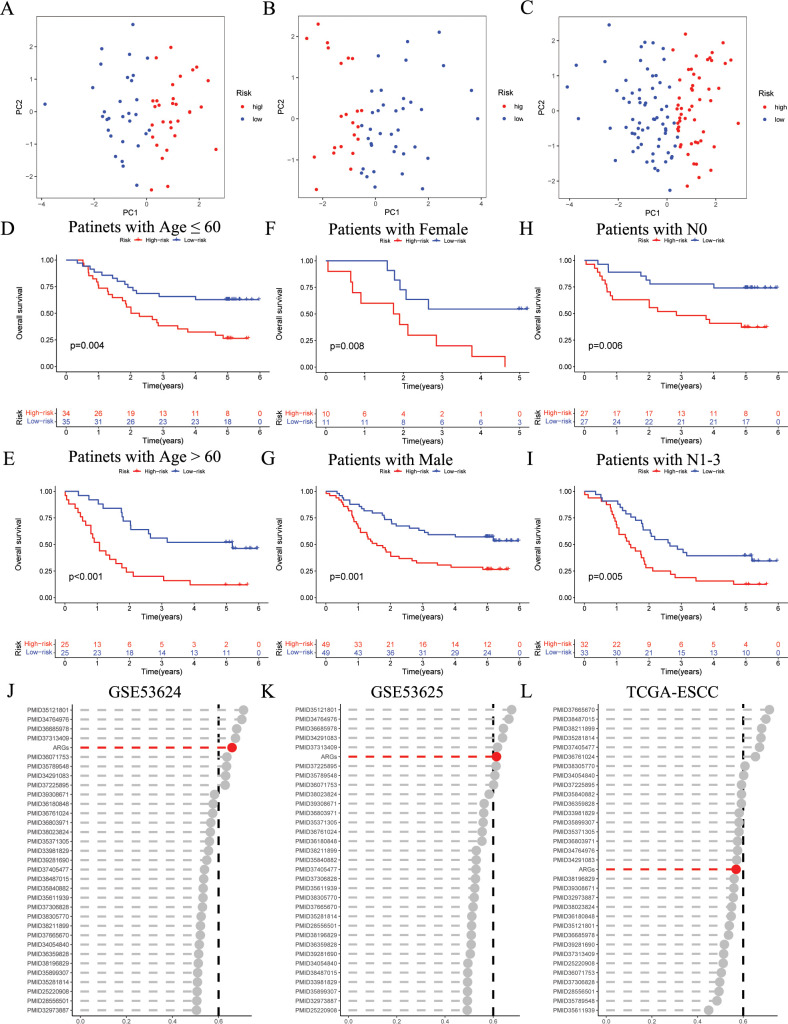
Evaluation of ARGs signature performance. **(A-C)** PCA analyses for the ARGs signature in the GSE53624 training (n = 60), testing (n = 59), and entire (n = 119) cohorts. **(D-I)** Kaplan–Meier curves of OS according to the ARGs score in the GSE53624 subgroup **(D)** patients with Age ≤ 60 years, *p* = 0.004; **(E)** patients with Age > 60 years, *p* < 0.001; **(F)** patients with Female, *p* = 0.008; **(G)** patients with Male, *p* = 0.001; **(H)** patients with N0, *p* = 0.006; **(I)** patients with N1-3, *p* = 0.005, with low ARGs group showing better outcomes. **(J-L)** C-index analysis ARGs and 32 published signatures in GSE53624 (n = 119), GSE53625 (n = 179), and TCGA-ESCC (n = 93) cohorts. ESCC, esophageal squamous cell carcinoma; ARGs, anoikis-related genes; PCA, principal component analysis; OS, overall survival.

The PCA results indicate that the ARGs signature has good grouping effect (**Figures 4A–C**). In addition, to explore the influence of the ARGs signature on patient prognosis in different clinical subgroups, we assessed the prognostic signature within various clinical features of ESCC. As shown in **Figures 4D–I**, in Age, Gender, and N subgroups, the low-risk ESCC group exhibited a higher survival rate than the high-risk ESCC group. Furthermore, to further validate the prognostic performance of the ARGs signature, we incorporated 32 published prognostic signatures and compared the C-index in the GSE53624, GSE53625, and TCGA-ESCC cohorts (**Figures 4J–L**). Our ARGs signature outperformed the majority of other published signatures in the GSE53624 and GSE53625 cohorts, and showed an intermediate performance in the TCGA-ESCC cohort.

**Figure 5:**
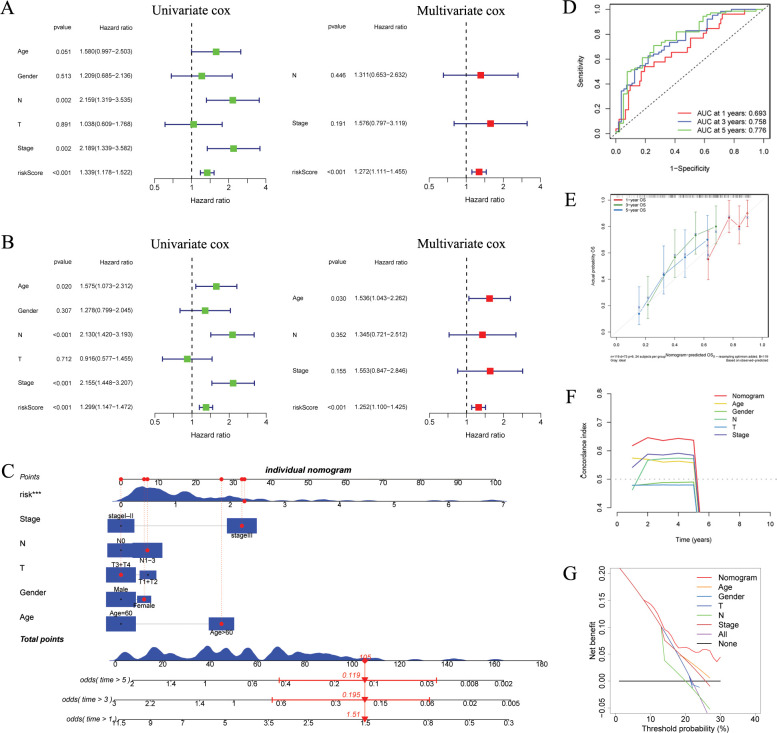
Independent prognostic analysis and construction of a nomogram. **(A, B)** Based on univariate and multivariate Cox analysis, ARGs was an independent prognostic risk factor in the GSE53624 [**(A)**, n = 119] and GSE53625 [**(B)**, n = 179] cohorts. **(C)** The ARGs-based nomogram considering patients’ other clinical features. **(D)** ROC curves showing the prediction performance of the nomogram in 1, 3, and 5-year OS. **(E)** Calibration curve of the nomogram for 1, 3, and 5-year OS. **(F)** The comparison of the C index between the nomogram and other clinical features. **(G)** Decision curve analysis showing the net benefit by applying the nomogram and other clinical features. OS, overall survival; ARGs, anoikis-related genes; ROC, Receiver operating characteristic. ***p < 0.001.

We initiated univariate and multivariate Cox analyses on the GSE53624 and GSE53625 cohorts to analyze the prognostic importance of ARGs signature in relation to various clinical features. In GSE53624 cohort, as depicted in **Figures 5A**, N, stage, and risk score were identified as prognostic risk factors for ESCC patients through univariate regression Cox analysis. Subsequently, multivariate Cox regression analysis revealed risk score (*p* < 0.001) as an independent prognostic risk factor for ESCC patients. Furthermore, in the GSE53625 cohort (**Figure 5B**), we observed that risk score continued to be an independent prognostic risk factor, indicating its robust prognostic ability in ESCC patients.

We developed a nomogram utilizing risk score and various clinical features (**Figure 5C**). ROC analysis revealed that nomogram exhibited a high level of specificity and sensitivity (**Figure 5D**). The calibration curve showed high consistency between the findings of the nomogram and the observed probability of OS in practical application (**Figure 5E**). The results from the C index and DCA indicated that the nomogram has a more robust and strong predictive capability as well as net clinical benefit than other clinical features (**Figures 5F, G**), indicating that this nomogram has the potential to be utilized as a precise prognostic tool for ESCC patients.

### 3.4 Different tumor-associated pathways between groups

**Figure 6:**
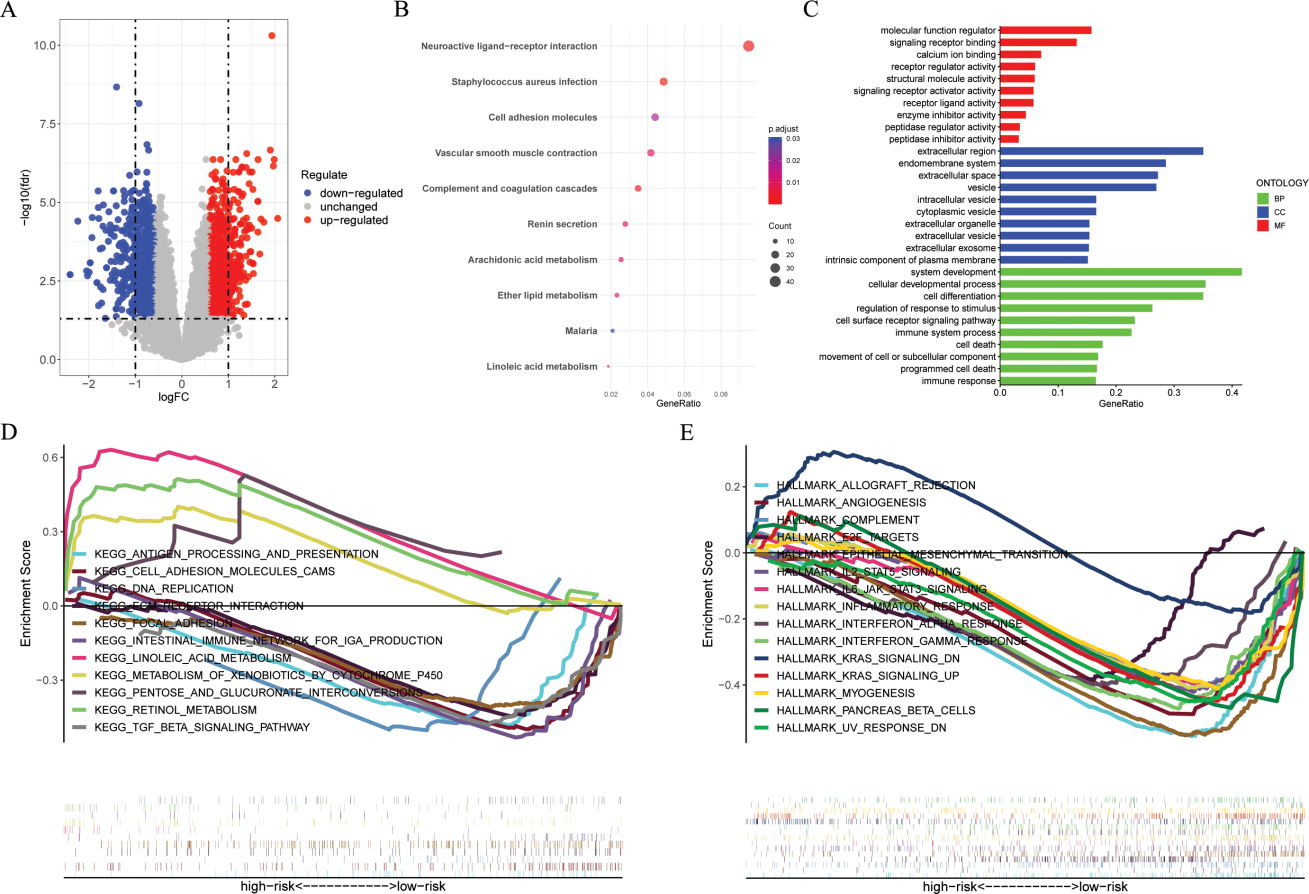
Functional enrichment analyses. **(A)** The volcano plot showing the DEGs between the high- and low-risk groups in the GSE53624 cohort (n = 119) with criteria of |logFC| > 0.585 and FDR < 0.05. **(B, C)** KEGG and GO enrichment analyses revealing the potential pathways enriched by the DEGs between the high- and low-risk groups. **(D, E)** GSEA enrichment analysis demonstrating the enrichment of differential genes to KEGG and Hallmark pathways between high- and low-risk groups. DEGs, different expression genes. KEGG, Kyoto Encyclopedia of Genes and Genomes; GO, Gene Ontology; GSEA, Gene Set Enrichment Analysis.

**Figure 6A** and **Supplementary Table S3** presents the DEGs between risk groups. KEGG pathway analysis indicated that DEGs were predominantly associated with “Cell adhesion molecules”, “Wnt signaling pathway”, and “Cytokine-cytokine receptor interaction” (**Figure 6B**). GO analysis, especially in the field of biological process (BP), indicated that there was a notable enrichment in terms of “immune system process”, “cell death”, “programmed cell death”, and “immune response” (**Figure 6C**). Furthermore, the GSEA analysis indicated a notable difference between subgroups (**Figures 6D, E**). According to “c2.cp.kegg_legacy.v2024.1.Hs.symbols.gmt” and “H.all.v2024.1.Hs.symbols.gmt”, we found that the high-risk group primarily showed activation of various cancer-related and immune-related signaling pathways, such as

“HALLMARK_WNT_BETA_CATENIN_SIGNALING“, "HALLMARK_KRAS_SIGNALING_UP",

"KEGG_ANTIGEN_PROCESSING_AND_PRESENTATION",“KEGG_TGF_BETA_SIGNALING_PATHWAY“, and “KEGG_INTESTINAL_IMMUNE_NETWORK_FOR_IGA_PRODUCTION“, and the low-risk group mainly exhibits activation of the following signaling pathways, such as “KEGG LINOLEIC ACID METABOLISM“, “KEGG RETINOL METABOLISM“, and “HALLMARK_KRAS_SIGNALING_DN“.

### 3.5 Comparison of tumor microenvironment between groups

**Figure 7:**
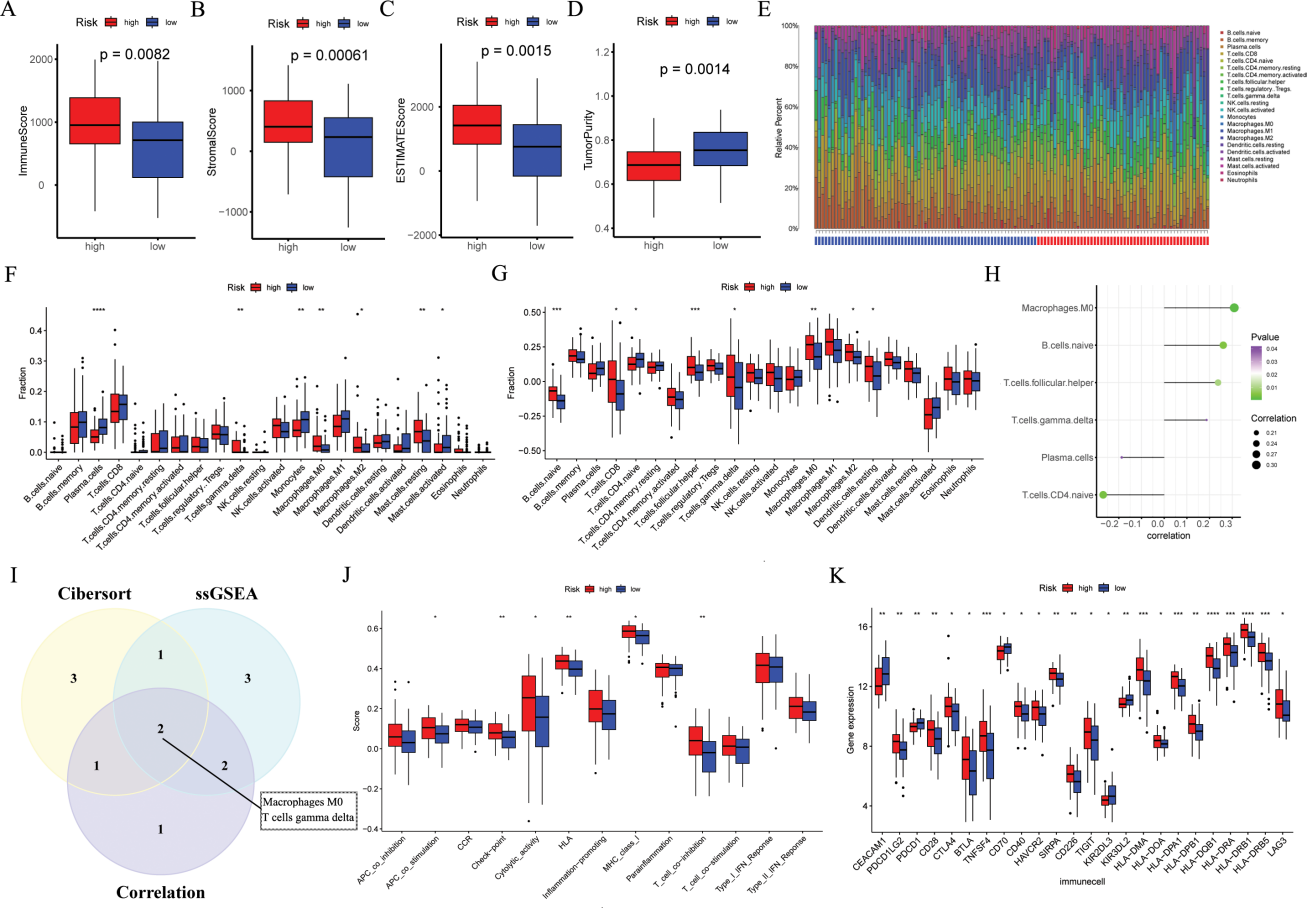
The immune landscape associated with the ARGs signature in ESCC. In the GSE53624 cohort (n = 119), **(A–D)** the immune score, stromal score, estimate score, and tumor purity were applied to quantify the different immune statuses between the high- and low-risk groups. **(E, F)** The CIBERSORT algorithm was used to evaluate differences in the abundances of 22 types of immune cells between the high- and low-risk groups. **(G)** The ssGSEA algorithm was used to analyze differences in 22 types of immune cells between the high- and low-risk groups. **(H)** Pearson correlation analysis was performed to assess the correlations between TME-infltrated cells and risk scores. **(I)** Venn plot showing the intersecting TME-infltrated cell types of CIBERSORT algorithm, ssGSEA algorithm, and correlation analysis. **(J)** The ssGSEA algorithm was used to analyze differences in immune functions between the high- and low-risk groups. **(K)** Box plot of expression difference of 24 immune checkpoints between the high- and low-risk groups. ESCC, esophageal squamous cell carcinoma; ARGs, anoikis-related genes; ssGSEA, single sample gene set enrichment analysis; TME, the tumor immune microenvironment. *p < 0.05, **p < 0.01, ***p < 0.001, ****p < 0.0001.

According to the Wilcoxon test, high-risk ESCC patients showed significantly higher immune, estimate, and stromal scores, as well as lower tumor purity scores (**Figures 7A–D**). According to the findings in the cibersort (**Figures 7E, F**), macrophages M0, macrophages M2, mast cell resting, and T cell gamma delta were more abundant in high-risk ESCC patients, while in the low-risk group, plasma cells, monocytes, and mast cell activated were more abundant. By applying the ssGSEA algorithm, we found significant differences between the two risk groups (**Figure 7G**). Among them, macrophages M0, macrophages M2, and T cell gamma delta were more abundant in high-risk ESCC patients, which is consistent with previous results. Besides, we employed Pearson correlation analysis to identify 6 immune cell types that are significantly correlated with risk scores (*p* < 0.05, **Figure 7H**). Ultimately, we identified two intersecting tumor microenvironment cell types (macrophages M0 and T cells gamma delta, **Figure 7I**). Next, we obtained immune function scores using the ssGSEA algorithm. Compared with low-risk ESCC patients, high-risk ESCC samples exhibited greater enrichment in co stimulation of antigen-presenting cells (APCs), check point, cytolytic activity, HLA, MHC class I, and T cell co-inhibition (**Figure 7J**). Finally, we compared the immune checkpoints (ICs) between the high and low groups, and found that 24 ICs had significant differences between the two groups (Wilcox test, *p* < 0.05, **Figure 7K**).

### 3.6 Comparison of TMB and immunotherapy response between groups

**Figure 8:**
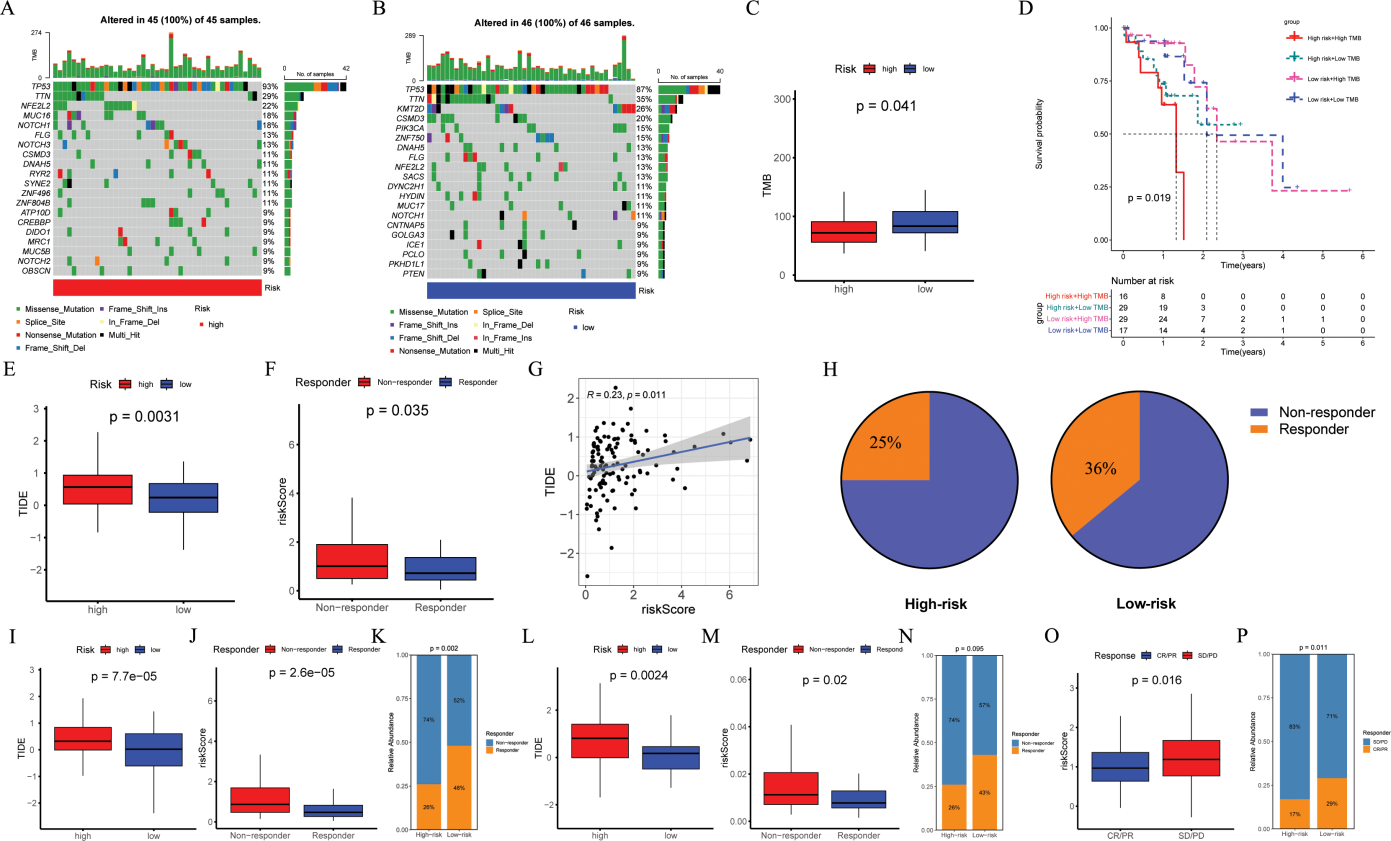
Evaluation of TMB and immunotherapy response. **(A, B)** The waterfall plot of the somatic mutation landscape in high- and low-risk patients in the TCGA-ESCC cohort (n = 93). **(C)** Boxplots of the difference in TMB between high- and low-risk groups (*p* = 0.041). **(D)** The Kaplan-Meier survival curve showing different overall survival (*p* = 0.019) among four subgroups (high-risk and high-TMB, high-risk and low-TMB, low-risk and high-TMB, low-risk and low-TMB). **(E, I, L)** Boxplots of the difference in TIDE between the high- and low-risk groups across GSE53624 (n = 119, *p* = 0.0031), GSE53625 (n = 179, *p* = 0.000075), and TCGA-ESCC (*p* = 0.024) cohorts. **(F, J, M)** Boxplots of the difference in risk score between non-response and response groups across GSE53624 (*p* = 0.035), GSE53625 (*p* = 0.000026), and TCGA-ESCC (*p* = 0.02) cohorts. **(G)** In GSE53624 cohort, the scatter plot of correlation between risk score and TIDE. **(H)** In GSE53624 cohort, percentages of immunotherapy responders in the high-risk group compared to the low-risk group. **(K, N)** Bar plots showing the proportion of immunotherapy response in the high- and low-risk groups across GSE53625 (*p* = 0.002) and TCGA-ESCC (*p* = 0.095) cohorts. **(O)** Boxplots of the difference in risk score between CR/PR and SD/PD groups in the IMvigor210 cohort (n = 298, *p* = 0.016). **(P)** Bar plots showing the proportion of immunotherapy response in the high- and low-risk groups in the IMvigor210 cohort (*p* = 0.011). Pearson correlation analysis was performed to assess the correlations between risk score and TIDE. Differences in immunotherapy response between high- and low-risk groups were compared using the chi-square test. Differences in TIDE and risk score between the two groups were analyzed using a student t-test. TMB, tumor mutational burden; ESCC, esophageal squamous cell carcinoma; TIDE, tumor immune dysfunction and exclusion.

In order to investigate the TMB between different risk groups, we conducted a mutation landscape within TCGA-ESCC patients (**Figures 8A, B**). Our investigation identified different mutation lineages in the high- and low-risk groups. For example, tumor suppressor genes NFE2L2 and NOTCH1 exhibited a higher frequency of mutations in the high-risk group at 22% and 18%, respectively, compared to 13% and 11% in the low-risk group. Moreover, an exploration into the association between risk scores and TMB revealed that individuals classified in the low-risk category exhibited elevated levels of TMB (**Figure 8C**, *p* = 0.041). When combining TMB with risk scores, it was observed that patients in the "high risk + high TMB" category experienced poorer outcomes (**Figure 8D**, *p* = 0.019).

To assess the potential of immunotherapy responses based on ARGs signature, we utilized the TIDE. Analysis of GSE53624, GSE53625, and TCGA-ESCC cohorts revealed higher TIDE scores in high-risk ESCC patients (*p* = 0.0031, **Figure 8E**; *p* < 0.001, **Figure 8I**; *p* = 0.0024, **Figure 8L**), with the mean risk score being significantly elevated in the non-response group compared to the response group (*p* = 0.035, **Figure 8F**; *p* < 0.001, **Figure 8J**; *p* = 0.02, **Figure 8M**). In the GSE53624 cohort, Pearson correlation analysis demonstrated a positive association between risk score and TIDE (**Figure 8G**, R = 0.23, *p* = 0.011), with a higher proportion of low-risk ESCC patients responding to immunotherapy (36%) than high-risk ESCC patients (25%) as shown in **Figure 8H**. Furthermore, compared to individuals in the high-risk group identified by risk score, analysis of both GSE53625 and TCGA-ESCC cohorts indicated a greater percentage of immunotherapy recipients in the low-risk group (**Figures 8K, N**). We further validated the predictive capability of ARGs signature for ICI responses using IMvigor210 cohort by calculating a risk score for each patient based on the coefficients and expression of the four modeling genes. Patients were then categorized into high-risk or low-risk groups according to their median risk score. Reassuringly, we observed that the average risk score was higher in patients belonging to the SD/PD group compared to those in the CR/PR group (**Figure 8O**, *p* = 0.016), and a lower proportion of high-risk patients achieved CR/PR compared to low-risk patients (**Figure 8P**, *p* = 0.011).

### 3.7 Drug sensitivity analysis

**Figure 9:**
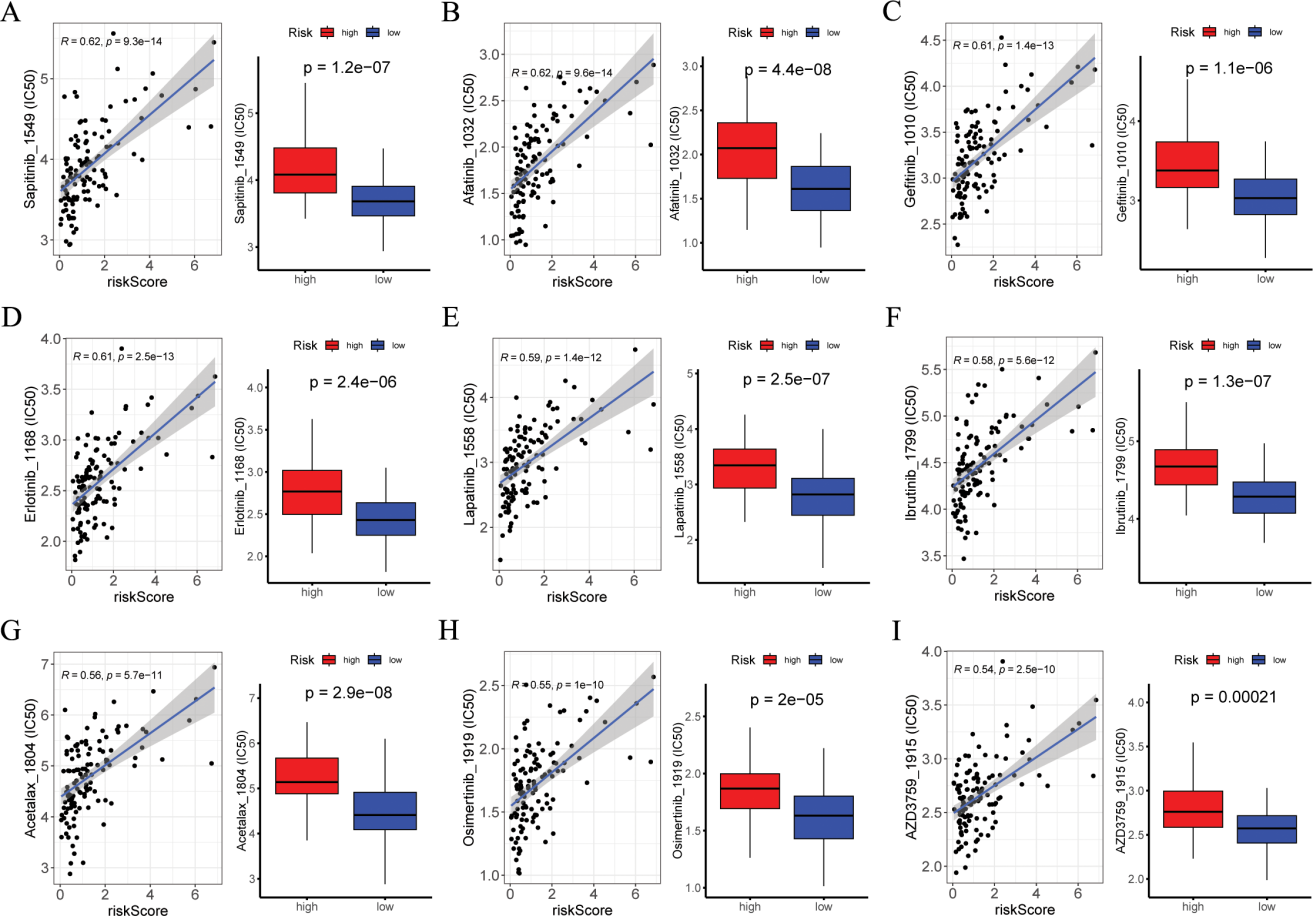
Exploration of drug compounds targeting the ARGs. In the GSE53624 cohort (n = 119), **(A-I)** correlation scatter plot of IC50 of the top 9 candidate drugs and risk score, and boxplots of the difference in IC50 of candidate drugs between high- and low-risk groups, with statistical significance assessed via the Wilcoxon rank sum test. Pearson correlation analysis was performed to assess the correlations between risk score and candidate drugs. ARGs, anoikis-related genes; IC50, the half-maximal inhibitory concentration.

This involved correlating IC50 values with risk score of various drugs and comparing drug sensitivity scores between risk groups. **Figures 9A–I** displayed the nine compounds with the most significant correlation between IC50 values and risk score, as determined by Pearson correlation and Wilcox tests (*p* < 0.05).

### 3.8 Single-cell sequencing data analysis

**Figure 10:**
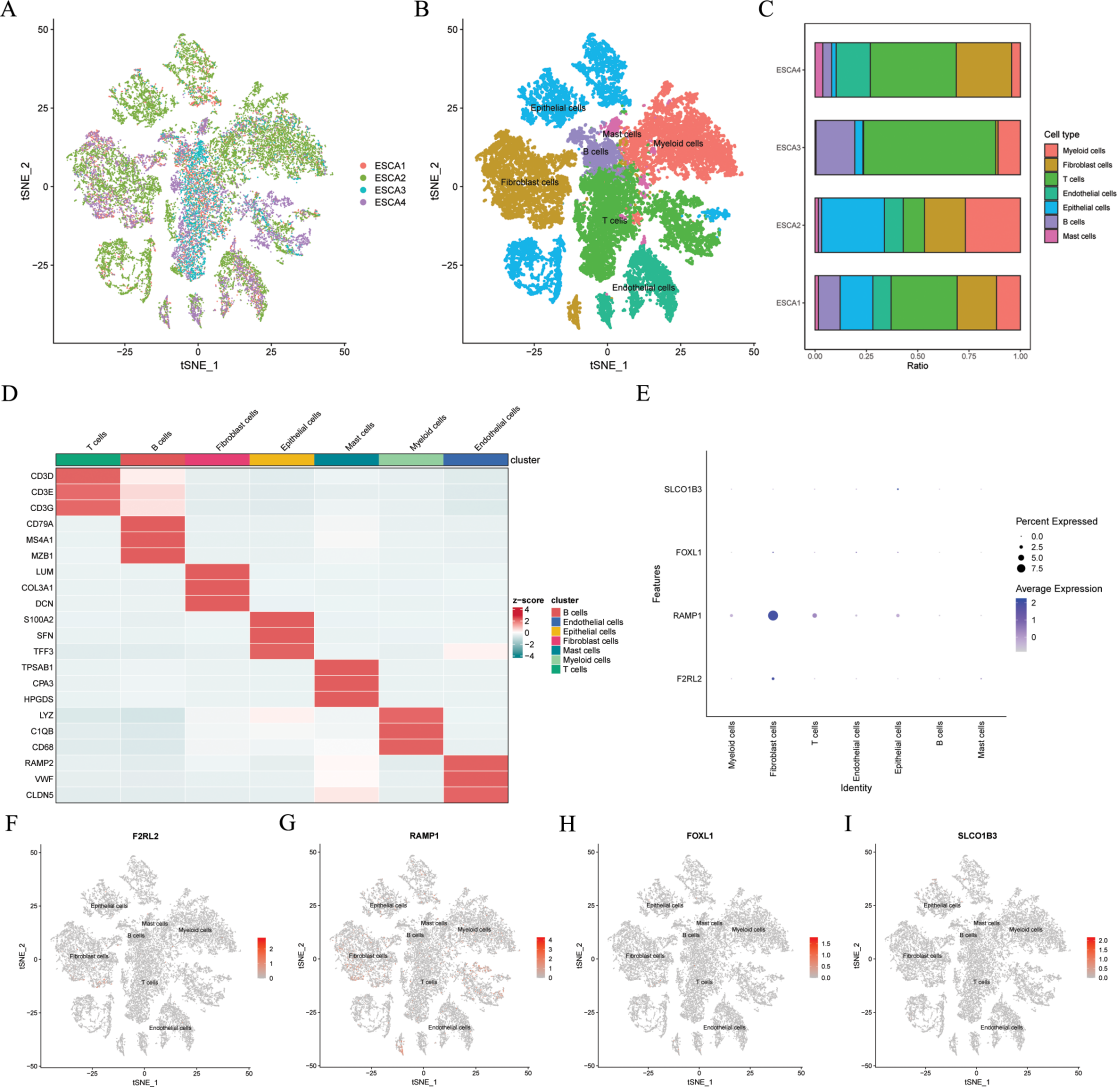
Gene expression distribution of ARGs on distinct cell types on single cell level. In the GSE188900 dataset (n=4), **(A)** tSNE plot of cell distribution in 4 patients with ESCC. **(B)** tSNE plot of 7 cell populations after dimension reduction. **(C)** Proportion of each cell population in different samples. **(D)** Heatmap showing the top 3 unique marker genes in each cellular subpopulation. **(E-I)** The four modeling genes levels in each cellular subpopulation. ESCC, esophageal squamous cell carcinoma; ARGs, anoikis-related genes; t-SNE, t-distributed stochastic neighbor embedding.

To mitigate batch effects, we used the Harmony package to effectively integrate the four patients with ESCC (**Figure 10A**). Subsequently, the top 2000 variant genes underwent dimensionality reduction using principal component analysis and t-SNE. The cells were grouped into 26 clusters using a resolution of 1 during the clustering process. We categorized the cells into nine major clusters using marker genes for different cell types: myeloid cells, fibroblasts cells, T cells, endothelial cells, epithelial cells, B cells, and mast cells (**Figure 10B**). The heatmap shows the three most significant marker genes for every cell population (**Figure 10D**). Additionally, we calculated the proportions of cell clusters in each sample and presented the results as histograms (**Figure 10C**). Furthermore, we analyzed the expression patterns of the four modeling genes in various cell types (**Figures 10E–I**). The results indicated the RAMP1 and F2RL2 were predominantly expressed in fibroblasts cells, while SLCO1B3 and FOXL1 had lower expression levels in various cells.

### 3.9 F2RL2 is an anoikis-related biomarker of ESCC

**Figure 11:**
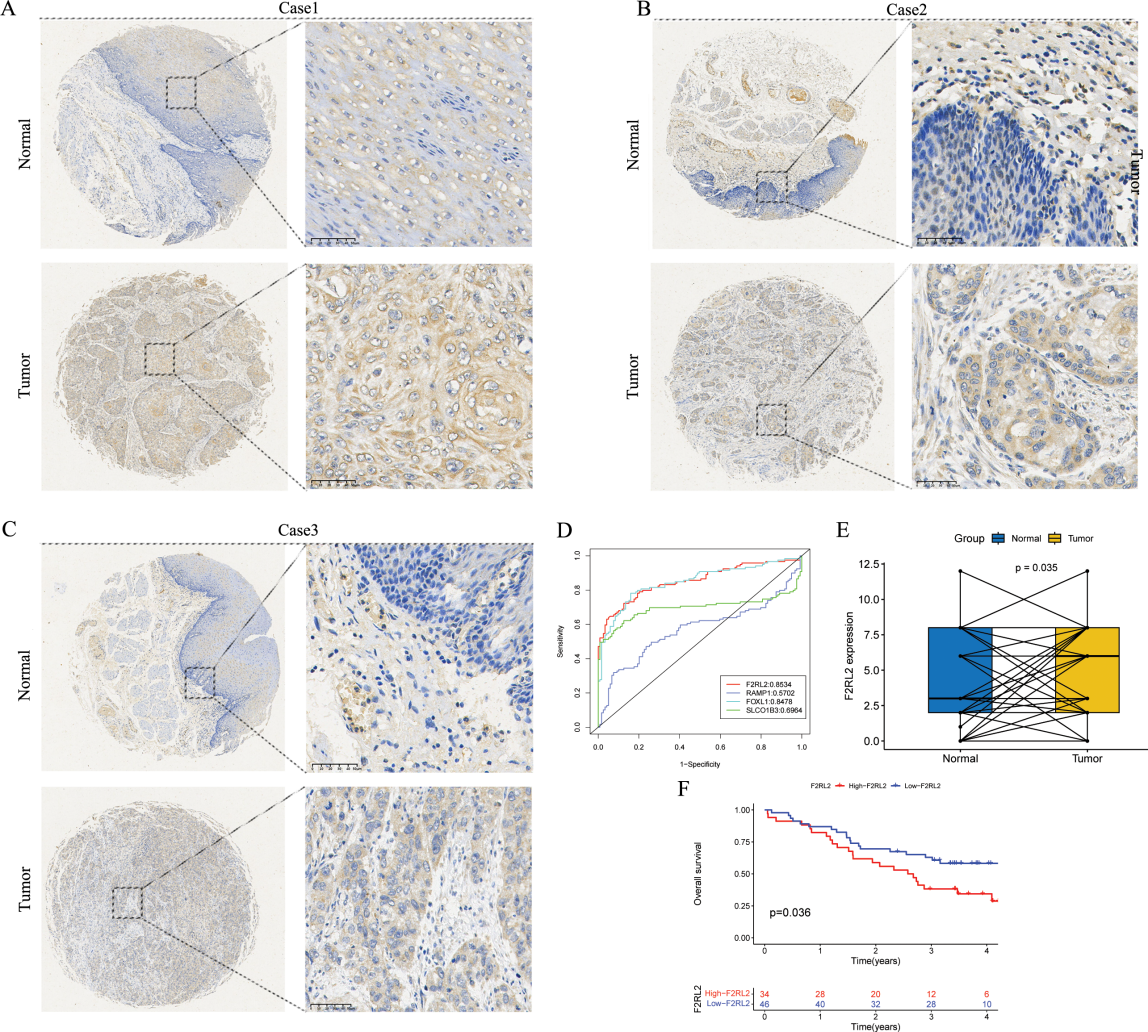
F2RL2 is an anoikis-related prognostic biomarker of ESCC. **(A-C)** Typical images of IHC staining with anti-F2RL2 antibody in paired ESCC tissues and adjacent normal tissues. **(D)** The four modeling genes were analyzed with the pROC package and visualized with the ggplot2 package. **(E)** Box plot of F2RL2 expression in 80 pairs of paired ESCC tissues and adjacent normal tissues (Paired t-test, *p* = 0.035). **(F)** The Kaplan-Meier survival curve showing different OS between the high- and low-F2RL2 groups. ESCC, esophageal squamous cell carcinoma; IHC, Immunohistochemistry; OS, overall survival.

The findings indicated that F2RL2 exhibited superior accuracy in predicting tumor status (tumor versus normal) compared to other variables (**Figure 11D**). Consequently, we proceeded with further validation of F2RL2 in ESCC. We collected 80 pairs of ESCC tissues and adjacent normal esophageal tissues for IHC staining. The protein level of F2RL2 in ESCC tissues was significantly higher than that in adjacent non-tumor tissues (**Figures 11A–C**). Paired t-test results indicated that the average expression of F2RL2 in ESCC tissues was higher compared to adjacent normal tissues (**Figure 11E**, *p* = 0.035). Based on the median expression of F2RL2, the 80 samples were stratified into high- and low-F2RL2 expression groups. KM survival analysis revealed that the high-F2RL2 expression groups exhibited a significantly poorer prognosis (*p* = 0.036, **Figure 11F**). These findings suggested that F2RL2 could serve as a valuable prognostic biomarker related to anoikis in ESCC.

## 4 Discussion

The treatment options for patients with ESCC, particularly those with advanced ESCC, have been significantly broadened by the emergence of immunotherapy. However, the prognosis for ESCC patients remains unfavorable due to the intricate and highly heterogeneous nature of ESCC tumors, as well as the absence of reliable prognostic biomarkers to indicate disease severity or predict response to immunotherapy (8, 9, 35, 57, 58). Anoikis, a distinct form of programmed cell death, plays a crucial role in cancer progression and metastasis (37–39), which may offer potential novel therapeutic strategies for anti-tumor treatment. Recently, a plethora of studies have demonstrated the significant value of ARGs prognostic signature in predicting the prognosis and immunotherapy response among cancer patients, including those with bladder cancer (48, 59), osteosarcoma (60), glioblastoma (46), colorectal cancer (61), clear cell renal cell carcinoma (62), liver hepatocellular carcinoma (63), and other malignancies. In this study, we employed multiple cohorts for comprehensive analysis, thoroughly investigated the expression patterns of ARGs in ESCC, and developed an ARGs prognostic signature. In comparison with 32 previously published prognostic features, the ARG signature surpassed most other published signatures in the GSE53624 and GSE53625 cohorts, and was at an intermediate level in the TCGA-ESCC cohorts. This feature was subsequently applied across various cohorts to forecast the prognosis of ESCC and assess the effectiveness of immunotherapy response, ultimately aiming to enhance the OS of patients with ESCC.

In this study, the ARGs signature consisted of four genes (RAMP1, FOXL1, SLCO1B3, and F2RL2) that have previously been documented to be closely linked with cancer. Receptor activity modifying protein 1 (RAMP1) serves as a co-receptor for specific G protein-coupled receptors, such as calcitonin gene-related peptide receptors, and the plasma membrane (64–66). In prostate cancer, a recent finding indicates that RAMP1 is a direct target gene of NKX3.1 and serves as a new biomarker (64). Additionally, in a study by Balood M. et al. (67), single-cell RNA sequencing analysis indicated that elevated RAMP1 expression associated with unfavorable clinical outcomes in melanoma patients. Moreover, Dallmayer M. et al. (68) demonstrated that the ablation of RAMP1 results in a reduction in clonal growth rate and tumorigenic potential of Ewing sarcoma cell lines. Furthermore, through bioinformatics analysis, Xie, L et al. (65) discovered RAMP1 could potentially be used as a biomarker for diagnosing and predicting the prognosis of osteosarcoma, and also as a molecular target for treating osteosarcoma. Forkhead box L1 (FOXL1), a member of the FOX superfamily (69, 70), is involved in cancer invasion and metastasis and shows abnormal expression in various tumors such as glioma (71), gallbladder cancer (70), pancreatic cancer (69), gastric cancer (72), and renal cancer (73). Similarly, the solute carrier organic anion transporter family member 1B3 (SLCO1B3) (74, 75), a functional transporter, plays a crucial role in the occurrence and development of tumors and has been found to be abnormally expressed in various tumors (75–80). Recent studies have also linked SLCO1B3 with resistance to anti-cancer treatments (75). Regulation factor II zombie receptor like 2 (F2RL2) is a G protein coupled receptor encoding PAR3 (81). Zhenhua Wu et al. (82) discovered that downregulation of F2RL2 expression can mitigate the damage caused by myocardial infarction. Furthermore, Mengnan Zhao et al. (83) identified heightened levels of F2RL2 expression in ESCC through immunofluorescence assay. Consistently, our IHC results revealed significantly elevated protein levels of F2RL2 in ESCC tissues compared to adjacent non-tumor tissues, and elevated F2RL2 expression was correlated with unfavorable prognosis. Our research findings indicated that F2RL2 serves as a valuable prognostic biomarker for ESCC.

TME plays a significant role in the development and evolution of tumors such as ESCC (84, 85). A thorough investigation of tumor infiltrating immune cells can clarify the potential mechanisms of cancer immune evasion and offer chances for the development of novel treatment strategies (25, 86). Our findings showed that the high-risk group exhibited a higher degree of immune infiltration compared with the low-risk group. Additional, substantial differences in immune checkpoint genes expression existed between the high- and low-risk groups. Considering the correlation between the expression levels of immune checkpoint genes and the efficacy of immunotherapy (25, 87), it can be inferred that this might be one of the reasons for the disparities in the immunotherapy response between the high- and low-risk groups. Furthermore, our findings revealed a significant elevation of M0 macrophages, M2 macrophages, and T cell gamma delta in the high-risk group. It is noteworthy that M0 macrophages have the capacity to differentiate into either M1 or M2 cells (88–90). M2 macrophages are recognized for their ability to suppress inflammatory responses in solid tumors, such as ESCC, and have various pro-tumor effects (91), and the accumulation of M2 macrophages is linked to a poor clinical prognosis (92–95). Furthermore, it has been documented that M2 macrophages exert a crucial role in ESCC by promoting the depletion of anti-tumor effector T cells in TME (30, 85, 96). Our study also confirmed that the high-risk group had a poorer response to immunotherapy than low-risk group, which we infer might be attributed to T cell exhaustion and immune escape mechanisms within the immunosuppressive TME. T cell gamma delta, which have alternative T cell receptor structures composed of gamma and delta chains, play a critical role in innate immunity by expanding their range of antigen recognition independently of MHC (97, 98). In the context of most cancer types, with only a few exceptions, T cell gamma delta is linked to a favorable prognosis (98–103). However, there is still uncertainty regarding the changes in subpopulations and functions of T cell gamma delta in ESCC, as well as their prognostic and diagnostic significance (97), necessitating further investigation in the future.

Immunotherapy is recognized as an effective and promising treatment modality, offering a novel approach to cancer therapy (8, 104). Nevertheless, only a specific proportion of patients benefit from immunotherapy, with an even smaller proportion experiencing sustained response (8, 105). Hence, precise prediction is essential for identifying the patients who will derive benefits from immunotherapy. Our research findings indicated that low-risk ESCC patients may exhibit a higher likelihood of positive response to immunotherapy, while high-risk patients are more inclined towards immune evasion. Furthermore, our analysis of the IMvigor210 cohort revealed that ARGs signature can effectively distinguish whether patients have immunotherapy responses. Moreover, considering the pivotal role of TMB in determining tumor response to immunotherapy, elevating TMB levels has the potential to augment the efficacy of immune checkpoint inhibitors (106–109). The findings of the study suggested that individuals at low risk show higher levels of TMB in comparison to those at high risk. It is important to note that individuals at low risk may demonstrate a stronger response to immunotherapy, which aligns with the aforementioned results. In conclusion, the validity of the constructed ARGs signature has been confirmed across multiple cohorts, including the IMvigor210, TCGA-ESCC, GSE53625, and GSE53624, underscoring the effectiveness of ARGs signature in predicting Immunotherapy response. However, the aforementioned conclusions are drawn from the analysis of RNA expression data acquired in public datasets. The scarcity of immunotherapy-related data in the ESCC cohort impedes a comprehensive evaluation of the influence of ARGs signature in predicting ESCC immunotherapy outcomes. In the future, it will be requisite to validate the efficacy of immunotherapy responses more extensively in genuine ESCC cohorts.

Although this study yields innovative and promising findings, certain limitations exist. Firstly, it is a retrospective study relying on public databases, featuring a limited sample size within the datasets. Validation in more diverse patient cohorts, multicenter studies, and real-world data is necessary in the future. Secondly, no additional experimental verification was carried out. In future research, further *in vitro* and *in vivo* investigations are necessary to validate this prognostic signature and explore the potential mechanisms underlying this signature. These issues warrant attention and need to be tackled in future research.

## 5 Conclusions

In conclusion, our research offers valuable insights into the expression patterns and roles of ARGs signature in ESCC. The ARGs signature serves as a robust predictor of prognosis and holds potential guiding significance in personalized clinical decision-making, particularly in the formulation of immunotherapy strategies for ESCC. Moving forward, there is a necessity for more extensive validation of the prognostic value and efficacy of immunotherapy response in real ESCC cohorts.

## Data Availability

The original contributions presented in the study are included in the article/**Supplementary Material**. Further inquiries can be directed to the corresponding author.

## References

[1] Morgan E Soerjomataram I Rumgay H Coleman HG Thrift AP Vignat J. The global landscape of esophageal squamous cell carcinoma and esophageal adenocarcinoma incidence and mortality in 2020 and projections to 2040: new estimates from GLOBOCAN 2020. Gastroenterology. (2022) 163:649–658.e2. doi: 10.1053/j.gastro.2022.05.054 35671803

[2] Erratum: Global cancer statistics. GLOBOCAN estimates of incidence and mortality worldwide for 36 cancers in 185 countries. CA Cancer J Clin. (2018) 70:313. doi: 10.3322/caac.21609 32767693

[3] Chen W Li H Ren J Zheng R Shi J Li J. Selection of high-risk individuals for esophageal cancer screening: A prediction model of esophageal squamous cell carcinoma based on a multicenter screening cohort in rural China. Int J Cancer. (2021) 148:329–39. doi: 10.1002/ijc.v148.2 32663318

[4] Deboever N Jones CM Yamashita K Ajani JA Hofstetter WL. Advances in diagnosis and management of cancer of the esophagus. Bmj. (2024) 385:e074962. doi: 10.1136/bmj-2023-074962 38830686

[5] Sung H Ferlay J Siegel RL Laversanne M Soerjomataram I Jemal A. Global cancer statistics 2020: GLOBOCAN estimates of incidence and mortality worldwide for 36 cancers in 185 countries. CA Cancer J Clin. (2021) 71:209–49. doi: 10.3322/caac.21660 33538338

[6] The global, regional, and national burden of oesophageal cancer and its attributabl risk factors in 195 countries and territoriea 1990-2017: a systematic analysis for the Global Burden of Disease Study 2017. Lancet Gastroenterol Hepatol. (2020) 5:582–97. doi: 10.1016/s2468-1253(20)30007-8 PMC7232026 32246941

[7] Yan Y Feng X Li C Lerut T Li H. Treatments for resectabl esophageal cancer: from traditional systemic therapy to immunotherapy. Chin Med J (Engl). (2022) 135:2143–56. doi: 10.1097/CM9.0000000000002371 PMC9771193 36525602

[8] Yang H Li X Yang W. Advances in targeted therapy and immunotherapy for esophageal cancer. Chin Med J (Engl). (2023) 136:1910–22. doi: 10.1097/CM9.0000000000002768 PMC10431250 37403208

[9] Puhr HC Prager GW Ilhan-Mutlu A. How we treat esophageal squamous cell carcinoma. ESMO Open. (2023) 8:100789. doi: 10.1016/j.esmoop.2023.100789 36791637 PMC9958251

[10] Wu HX Pan YQ He Y Wang ZX Guan WL Chen YX. Clinical benefit of first-line programmed death-1 antibody plus chemotherapy in low programmed cell death ligand 1-expressing esophageal squamous cell carcinoma: A *post hoc* analysis of JUPITER-06 and meta-analysis. J Clin Oncol. (2023) 41:1735–46. doi: 10.1200/JCO.22.01490 PMC10022847 36473145

[11] Curran MA Montalvo W Yagita H Allison JP. PD-1 and CTLA-4 combination blockade expands infiltrating T cells and reduces regulatory T and myeloid cells within B16 melanoma tumors. Proc Natl Acad Sci U.S.A. (2010) 107:4275–80. doi: 10.1073/pnas.0915174107 PMC2840093 20160101

[12] Seiwert TY Burtness B Mehra R Weiss J Berger R Eder JP. Safety and clinical activity of pembrolizumab for treatment of recurrent or metastatic squamous cell carcinoma of the head and neck (KEYNOTE-012): an open-label, multicentre, phase 1b trial. Lancet Oncol. (2016) 17:956–65. doi: 10.1016/S1470-2045(16)30066-3 27247226

[13] Sangro B Sarobe P Hervás-Stubbs S Melero I. Advances in immunotherapy for hepatocellular carcinoma. Nat Rev Gastroenterol Hepatol. (2021) 18:525–43. doi: 10.1038/s41575-021-00438-0 PMC8042636 33850328

[14] Deleuze A Saout J Dugay F Peyronnet B Mathieu R Verhoest G. Immunotherapy in renal cell carcinoma: the future is now. Int J Mol Sci. (2020) 21(7). doi: 10.3390/ijms21072532 PMC7177761 32260578

[15] Shah MA Bennouna J Doi T Shen L Kato K Adenis A. KEYNOTE-975 study design: a Phase III study of definitive chemoradiotherapy plus pembrolizumab in patients with esophageal carcinoma. Future Oncol. (2021) 17:1143–53. doi: 10.2217/fon-2020-0969 PMC7927908 33533655

[16] Sun JM Shen L Shah MA Enzinger P Adenis A Doi T. Pembrolizumab plus chemotherapy versus chemotherapy alone for first-line treatment of advanced oesophageal cancer (KEYNOTE-590): a randomised, placebo-controlled, phase 3 study. Lancet. (2021) 398:759–71. doi: 10.1016/S0140-6736(21)01234-4 34454674

[17] Luo H Lu J Bai Y Mao T Wang J Fan Q. Effect of camrelizumab vs placebo added to chemotherapy on survival and progression-free survival in patients with advanced or metastatic esophageal squamous cell carcinoma: the ESCORT-1st randomized clinical trial. Jama. (2021) 326:916–25. doi: 10.1001/jama.2021.12836 PMC8441593 34519801

[18] Doki Y Ajani JA Kato K Xu J Wyrwicz L Motoyama S. Nivolumab combination therapy in advanced esophageal squamous-cell carcinoma. N Engl J Med. (2022) 386:449–62. doi: 10.1056/NEJMoa2111380 35108470

[19] Huang J Xu J Chen Y Zhuang W Zhang Y Chen Z. Camrelizumab versus investigator's choice of chemotherapy as second-line therapy for advanced or metastatic oesophageal squamous cell carcinoma (ESCORT): a multicentre, randomised, open-label, phase 3 study. Lancet Oncol. (2020) 21:832–42. doi: 10.1016/S1470-2045(20)30110-8 32416073

[20] Smyth EC Lagergren J Fitzgerald RC Lordick F Shah MA Lagergren P. Oesophageal cancer. Nat Rev Dis Primers. (2017) 3:17048. doi: 10.1038/nrdp.2017.48 28748917 PMC6168059

[21] Marshall HT Djamgoz MBA. Immuno-oncology: emerging targets and combination therapies. Front Oncol. (2018) 8:315. doi: 10.3389/fonc.2018.00315 30191140 PMC6115503

[22] Quail DF Joyce JA. Microenvironmental regulation of tumor progression and metastasis. Nat Med. (2013) 19:1423–37. doi: 10.1038/nm.3394 PMC3954707 24202395

[23] Wang W Zhao H Wang S. Identification of a novel immune-related gene signature for prognosis and the tumor microenvironment in patients with uveal melanoma combining single-cell and bulk sequencing data. Front Immunol. (2023) 14:1099071. doi: 10.3389/fimmu.2023.1099071 36793711 PMC9922847

[24] Ye B Jiang A Liang F Wang C Liang X Zhang P. Navigating the immune landscape with plasma cells: A pan-cancer signature for precision immunotherapy. Biofactors. (2025) 51:e2142. doi: 10.1002/biof.v51.1 39495620

[25] Zhang P Zhang X Cui Y Gong Z Wang W Lin S. Revealing the role of regulatory T cells in the tumor microenvironment of lung adenocarcinoma: a novel prognostic and immunotherapeutic signature. Front Immunol. (2023) 14:1244144. doi: 10.3389/fimmu.2023.1244144 37671160 PMC10476870

[26] Zhang L Cui Y Mei J Zhang Z Zhang P. Exploring cellular diversity in lung adenocarcinoma epithelium: Advancing prognostic methods and immunotherapeutic strategies. Cell Prolif. (2024) 57:e13703. doi: 10.1111/cpr.v57.11 38946232 PMC11533061

[27] Zhang L Cui Y Zhou G Zhang Z Zhang P. Leveraging mitochondrial-programmed cell death dynamics to enhance prognostic accuracy and immunotherapy efficacy in lung adenocarcinoma. J Immunother Cancer. (2024) 12(10). doi: 10.1136/jitc-2024-010008 PMC11529751 39455097

[28] Mariathasan S Turley SJ Nickles D Castiglioni A Yuen K Wang Y. TGFβ attenuates tumour response to PD-L1 blockade by contributing to exclusion of T cells. Nature. (2018) 554:544–8. doi: 10.1038/nature25501 PMC6028240 29443960

[29] Hui L Chen Y. Tumor microenvironment: Sanctuary of the devil. Cancer Lett. (2015) 368:7–13. doi: 10.1016/j.canlet.2015.07.039 26276713

[30] Zheng Y Chen Z Han Y Han L Zou X Zhou B. Immune suppressive landscape in the human esophageal squamous cell carcinoma microenvironment. Nat Commun. (2020) 11:6268. doi: 10.1038/s41467-020-20019-0 33293583 PMC7722722

[31] Dikiy S Rudensky AY. Principles of regulatory T cell function. Immunity. (2023) 56:240–55. doi: 10.1016/j.immuni.2023.01.004 36792571

[32] Qiu L Yue J Ding L Yin Z Zhang K Zhang H. Cancer-associated fibroblasts: An emerging target against esophageal squamous cell carcinoma. Cancer Lett. (2022) 546:215860. doi: 10.1016/j.canlet.2022.215860 35948121

[33] Ren Q Zhang P Zhang X Feng Y Li L Lin H. A fibroblast-associated signature predicts prognosis and immunotherapy in esophageal squamous cell cancer. Front Immunol. (2023) 14:1199040. doi: 10.3389/fimmu.2023.1199040 37313409 PMC10258351

[34] Sahai E Astsaturov I Cukierman E DeNardo DG Egeblad M Evans RM. A framework for advancing our understanding of cancer-associated fibroblasts. Nat Rev Cancer. (2020) 20:174–86. doi: 10.1038/s41568-019-0238-1 PMC7046529 31980749

[35] Xiong K Tao Z Zhang Z Wang J Zhang P. Identification and validation of a prognostic immune-related gene signature in esophageal squamous cell carcinoma. Front Bioeng Biotechnol. (2022) 10:850669. doi: 10.3389/fbioe.2022.850669 35497331 PMC9043362

[36] Yao J Duan L Huang X Liu J Fan X Xiao Z. Development and validation of a prognostic gene signature correlated with M2 macrophage infiltration in esophageal squamous cell carcinoma. Front Oncol. (2021) 11:769727. doi: 10.3389/fonc.2021.769727 34926275 PMC8677679

[37] Meredith JE Jr. Fazeli B Schwartz MA. The extracellular matrix as a cell survival factor. Mol Biol Cell. (1993) 4:953–61. doi: 10.1091/mbc.4.9.953 PMC275725 8257797

[38] Sattari Fard F Jalilzadeh N Mehdizadeh A Sajjadian F Velaei K. Understanding and targeting anoikis in metastasis for cancer therapies. Cell Biol Int. (2023) 47:683–98. doi: 10.1002/cbin.11970 36453448

[39] Taddei ML Giannoni E Fiaschi T Chiarugi P. Anoikis: an emerging hallmark in health and diseases. J Pathol. (2012) 226:380–93. doi: 10.1002/path.v226.2 21953325

[40] Wu Z Yu J Han T Tu Y Su F Li S. System analysis based on Anoikis-related genes identifies MAPK1 as a novel therapy target for osteosarcoma with neoadjuvant chemotherapy. BMC Musculoskelet Disord. (2024) 25:437. doi: 10.1186/s12891-024-07547-2 38835052 PMC11149263

[41] Eckhardt BL Cao Y Redfern AD Chi LH Burrows AD Roslan S. Activation of canonical BMP4-SMAD7 signaling suppresses breast cancer metastasis. Cancer Res. (2020) 80:1304–15. doi: 10.1158/0008-5472.CAN-19-0743 31941699

[42] Wang J Luo Z Lin L Sui X Yu L Xu C. Anoikis-associated lung cancer metastasis: mechanisms and therapies. Cancers (Basel). (2022) 14(19). doi: 10.3390/cancers14194791 PMC9564242 36230714

[43] Bose M Sanders A De C Zhou R Lala P Shwartz S. Targeting tumor-associated MUC1 overcomes anoikis-resistance in pancreatic cancer. Transl Res. (2023) 253:41–56. doi: 10.1016/j.trsl.2022.08.010 36031050

[44] Qi L Chen F Wang L Yang Z Zhang W Li ZH. Identification of anoikis-related molecular patterns to define tumor microenvironment and predict immunotherapy response and prognosis in soft-tissue sarcoma. Front Pharmacol. (2023) 14:1136184. doi: 10.3389/fphar.2023.1136184 36937870 PMC10014785

[45] Yang L Xu F. A novel anoikis-related risk model predicts prognosis in patients with colorectal cancer and responses to different immunotherapy strategies. J Cancer Res Clin Oncol. (2023) 149:10879–92. doi: 10.1007/s00432-023-04945-2 PMC11796863 37318595

[46] Sun Z Zhao Y Wei Y Ding X Tan C Wang C. Identification and validation of an anoikis-associated gene signature to predict clinical character, stemness, IDH mutation, and immune filtration in glioblastoma. Front Immunol. (2022) 13:939523. doi: 10.3389/fimmu.2022.939523 36091049 PMC9452727

[47] Zhang M Liang Y Song P. Identification of prognostic value of anoikis-related gene score model combined with tumor microenvironment score models in esophageal squamous cell carcinoma. J Gene Med. (2024) 26:e3624. doi: 10.1002/jgm.3624 38087999

[48] Xie T Peng S Liu S Zheng M Diao W Ding M. Multi-cohort validation of Ascore: an anoikis-based prognostic signature for predicting disease progression and immunotherapy response in bladder cancer. Mol Cancer. (2024) 23:30. doi: 10.1186/s12943-024-01945-9 38341586 PMC10858533

[49] Blanche P Dartigues JF Jacqmin-Gadda H. Estimating and comparing time-dependent areas under receiver operating characteristic curves for censored event times with competing risks. Stat Med. (2013) 32:5381–97. doi: 10.1002/sim.v32.30 24027076

[50] Joliffe IT Morgan BJ. Principal component analysis and exploratory factor analysis. Stat Methods Med Res. (1992) 1:69–95. doi: 10.1177/096228029200100105 1341653

[51] Ritchie ME Phipson B Wu D Hu Y Law CW Shi W. limma powers differential expression analyses for RNA-sequencing and microarray studies. Nucleic Acids Res. (2015) 43:e47. doi: 10.1093/nar/gkv007 25605792 PMC4402510

[52] Yu G Wang LG Han Y He QY. clusterProfiler: an R package for comparing biological themes among gene clusters. Omics. (2012) 16:284–7. doi: 10.1089/omi.2011.0118 PMC3339379 22455463

[53] Newman AM Liu CL Green MR Gentles AJ Feng W Xu Y. Robust enumeration of cell subsets from tissue expression profiles. Nat Methods. (2015) 12:453–7. doi: 10.1038/nmeth.3337 PMC4739640 25822800

[54] Mayakonda A Lin DC Assenov Y Plass C Koeffler HP. Maftools: efficient and comprehensive analysis of somatic variants in cancer. Genome Res. (2018) 28:1747–56. doi: 10.1101/gr.239244.118 PMC6211645 30341162

[55] Jiang P Gu S Pan D Fu J Sahu A Hu X. Signatures of T cell dysfunction and exclusion predict cancer immunotherapy response. Nat Med. (2018) 24:1550–8. doi: 10.1038/s41591-018-0136-1 PMC6487502 30127393

[56] Maeser D Gruener RF Huang RS. oncoPredict: an R package for predicting *in vivo* or cancer patient drug response and biomarkers from cell line screening data. Brief Bioinform. (2021) 22(6). doi: 10.1093/bib/bbab260 PMC8574972 34260682

[57] Lagergren J Lagergren P. Oesophageal cancer. Bmj. (2010) 341:c6280. doi: 10.1136/bmj.c6280 21112905

[58] Kakeji Y Oshikiri T Takiguchi G Kanaji S Matsuda T Nakamura T. Multimodality approaches to control esophageal cancer: development of chemoradiotherapy, chemotherapy, and immunotherapy. Esophagus. (2021) 18:25–32. doi: 10.1007/s10388-020-00782-1 32964312

[59] Dong Y Xu C Su G Li Y Yan B Liu Y. Clinical value of anoikis-related genes and molecular subtypes identification in bladder urothelial carcinoma and *in vitro* validation. Front Immunol. (2023) 14:1122570. doi: 10.3389/fimmu.2023.1122570 37275895 PMC10232821

[60] Zhang X Wen Z Wang Q Ren L Zhao S. A novel stratification framework based on anoikis-related genes for predicting the prognosis in patients with osteosarcoma. Front Immunol. (2023) 14:1199869. doi: 10.3389/fimmu.2023.1199869 37575253 PMC10413143

[61] Li C Weng J Yang L Gong H Liu Z. Development of an anoikis-related gene signature and prognostic model for predicting the tumor microenvironment and response to immunotherapy in colorectal cancer. Front Immunol. (2024) 15:1378305. doi: 10.3389/fimmu.2024.1378305 38779664 PMC11109372

[62] Liu Y Shi Z Zheng J Zheng Z Sun H Xuan Z. Establishment and validation of a novel anoikis-related prognostic signature of clear cell renal cell carcinoma. Front Immunol. (2023) 14:1171883. doi: 10.3389/fimmu.2023.1171883 37056778 PMC10086373

[63] Chen Y Huang W Ouyang J Wang J Xie Z. Identification of anoikis-related subgroups and prognosis model in liver hepatocellular carcinoma. Int J Mol Sci. (2023) 24(3). doi: 10.3390/ijms24032862 PMC9918018 36769187

[64] Logan M Anderson PD Saab ST Hameed O Abdulkadir SA. RAMP1 is a direct NKX3.1 target gene up-regulated in prostate cancer that promotes tumorigenesis. Am J Pathol. (2013) 183:951–63. doi: 10.1016/j.ajpath.2013.05.021 PMC3763771 23867798

[65] Xie L Xiao W Fang H Liu G. RAMP1 as a novel prognostic biomarker in pan-cancer and osteosarcoma. PloS One. (2023) 18:e0292452. doi: 10.1371/journal.pone.0292452 37796823 PMC10553254

[66] Russell FA King R Smillie SJ Kodji X Brain SD. Calcitonin gene-related peptide: physiology and pathophysiology. Physiol Rev. (2014) 94:1099–142. doi: 10.1152/physrev.00034.2013 PMC4187032 25287861

[67] Balood M Ahmadi M Eichwald T Ahmadi A Majdoubi A Roversi K. Nociceptor neurons affect cancer immunosurveillance. Nature. (2022) 611:405–12. doi: 10.1038/s41586-022-05374-w PMC9646485 36323780

[68] Dallmayer M Li J Ohmura S Alba Rubio R Baldauf MC Hölting TLB. Targeting the CALCB/RAMP1 axis inhibits growth of Ewing sarcoma. Cell Death Dis. (2019) 10:116. doi: 10.1038/s41419-019-1372-0 30741933 PMC6370763

[69] Zhang G He P Gaedcke J Ghadimi BM Ried T Yfantis HG. FOXL1, a novel candidate tumor suppressor, inhibits tumor aggressiveness and predicts outcome in human pancreatic cancer. Cancer Res. (2013) 73:5416–25. doi: 10.1158/0008-5472.CAN-13-0362 PMC3766408 23801748

[70] Qin Y Gong W Zhang M Wang J Tang Z Quan Z. Forkhead box L1 is frequently downregulated in gallbladder cancer and inhibits cell growth through apoptosis induction by mitochondrial dysfunction. PloS One. (2014) 9:e102084. doi: 10.1371/journal.pone.0102084 25010679 PMC4092092

[71] Chen A Zhong L Lv J. FOXL1 overexpression is associated with poor outcome in patients with glioma. Oncol Lett. (2019) 18:751–7. doi: 10.3892/ol.2019.10351 PMC6540307 31289550

[72] Ertao Z Jianhui C Chuangqi C Changjiang Q Sile C Yulong H. Low level of FOXL1 indicates a worse prognosis for gastric cancer patients. Tumour Biol. (2016) 37:11331–7. doi: 10.1007/s13277-016-4890-8 26960689

[73] Yang FQ Yang FP Li W Liu M Wang GC Che JP. Foxl1 inhibits tumor invasion and predicts outcome in human renal cancer. Int J Clin Exp Pathol. (2014) 7(1):110–22. PMC3885465 24427331

[74] McFeely SJ Ritchie TK Yu J Nordmark A Levy RH Ragueneau-Majlessi I. Identification and evaluation of clinical substrates of organic anion transporting polypeptides 1B1 and 1B3. Clin Transl Sci. (2019) 12:379–87. doi: 10.1111/cts.12623 PMC6662428 30706983

[75] Sun R Ying Y Tang Z Liu T Shi F Li H. The emerging role of the SLCO1B3 protein in cancer resistance. Protein Pept Lett. (2020) 27:17–29. doi: 10.2174/0929866526666190926154248 31556849 PMC6978646

[76] Hase H Aoki M Matsumoto K Nakai S Nagata T Takeda A. Cancer type−SLCO1B3 promotes epithelial−mesenchymal transition resulting in the tumour progression of non−small cell lung cancer. Oncol Rep. (2021) 45:309–16. doi: 10.3892/or.2020.7839 33155667

[77] Tang T Wang G Liu S Zhang Z Liu C Li F. Highly expressed SLCO1B3 inhibits the occurrence and development of breast cancer and can be used as a clinical indicator of prognosis. Sci Rep. (2021) 11:631. doi: 10.1038/s41598-020-80152-0 33436824 PMC7803962

[78] Zhi L Zhao L Zhang X Liu W Gao B Wang F. SLCO1B3 promotes colorectal cancer tumorigenesis and metastasis through STAT3. Aging (Albany NY). (2021) 13:22164–75. doi: 10.18632/aging.203502 PMC8507254 34526411

[79] Lee W Belkhiri A Lockhart AC Merchant N Glaeser H Harris EI. Overexpression of OATP1B3 confers apoptotic resistance in colon cancer. Cancer Res. (2008) 68:10315–23. doi: 10.1158/0008-5472.CAN-08-1984 PMC2605661 19074900

[80] Alam K Farasyn T Ding K Yue W. Characterization of liver- and cancer-type-organic anion transporting polypeptide (OATP) 1B3 messenger RNA expression in normal and cancerous human tissues. Drug Metab Lett. (2018) 12:24–32. doi: 10.2174/1872312812666180326110146 29577869 PMC6133766

[81] Hänzelmann S Wang J Güney E Tang Y Zhang E Axelsson AS. Thrombin stimulates insulin secretion via protease-activated receptor-3. Islets. (2015) 7:e1118195. doi: 10.1080/19382014.2015.1118195 26742564 PMC4878264

[82] Wu Z Geng J Bai Y Qi Y Chang C Jiao Y. miR-125b-5p alleviates the damage of myocardial infarction by inhibiting the NFAT2 to reduce F2RL2 expression. Regener Med. (2023) 18:543–59. doi: 10.2217/rme-2022-0150 37340944

[83] Zhao M Jin X Chen Z Zhang H Zhan C Wang H. Weighted correlation network analysis of cancer stem cell-related prognostic biomarkers in esophageal squamous cell carcinoma. Technol Cancer Res Treat. (2022) 21:15330338221117003. doi: 10.1177/15330338221117003 35899307 PMC9340319

[84] Nallasamy P Nimmakayala RK Parte S Are AC Batra SK Ponnusamy MP. Tumor microenvironment enriches the stemness features: the architectural event of therapy resistance and metastasis. Mol Cancer. (2022) 21:225. doi: 10.1186/s12943-022-01682-x 36550571 PMC9773588

[85] Zhang J Dong Y Di S Xie S Fan B Gong T. Tumor associated macrophages in esophageal squamous carcinoma: Promising therapeutic implications. BioMed Pharmacother. (2023) 167:115610. doi: 10.1016/j.biopha.2023.115610 37783153

[86] Zhang Y Zhang Z. The history and advances in cancer immunotherapy: understanding the characteristics of tumor-infiltrating immune cells and their therapeutic implications. Cell Mol Immunol. (2020) 17:807–21. doi: 10.1038/s41423-020-0488-6 PMC7395159 32612154

[87] Ahluwalia P Ahluwalia M Mondal AK Sahajpal N Kota V Rojiani MV. Immunogenomic gene signature of cell-death associated genes with prognostic implications in lung cancer. Cancers (Basel). (2021) 13(1). doi: 10.3390/cancers13010155 PMC7795632 33466402

[88] Wang Y Lyu Z Qin Y Wang X Sun L Zhang Y. FOXO1 promotes tumor progression by increased M2 macrophage infiltration in esophageal squamous cell carcinoma. Theranostics. (2020) 10:11535–48. doi: 10.7150/thno.45261 PMC7546008 33052231

[89] Wang C Ma C Gong L Guo Y Fu K Zhang Y. Macrophage polarization and its role in liver disease. Front Immunol. (2021) 12:803037. doi: 10.3389/fimmu.2021.803037 34970275 PMC8712501

[90] Xia T Zhang M Lei W Yang R Fu S Fan Z. Advances in the role of STAT3 in macrophage polarization. Front Immunol. (2023) 14:1160719. doi: 10.3389/fimmu.2023.1160719 37081874 PMC10110879

[91] Chaintreuil P Kerreneur E Bourgoin M Savy C Favreau C Robert G. The generation, activation, and polarization of monocyte-derived macrophages in human Malignancies. Front Immunol. (2023) 14:1178337. doi: 10.3389/fimmu.2023.1178337 37143666 PMC10151765

[92] Zhao Y Sun J Li Y Zhou X Zhai W Wu Y. Tryptophan 2,3-dioxygenase 2 controls M2 macrophages polarization to promote esophageal squamous cell carcinoma progression via AKT/GSK3β/IL-8 signaling pathway. Acta Pharm Sin B. (2021) 11:2835–49. doi: 10.1016/j.apsb.2021.03.009 PMC8463272 34589400

[93] Xu Y Liao C Liu R Liu J Chen Z Zhao H. IRGM promotes glioma M2 macrophage polarization through p62/TRAF6/NF-κB pathway mediated IL-8 production. Cell Biol Int. (2019) 43:125–35. doi: 10.1002/cbin.11061 PMC13397409 30288851

[94] Xu Y Cui G Jiang Z Li N Zhang X. Survival analysis with regard to PD-L1 and CD155 expression in human small cell lung cancer and a comparison with associated receptors. Oncol Lett. (2019) 17:2960–8. doi: 10.3892/ol.2019.9910 PMC6365950 30854074

[95] Cassetta L Pollard JW. Targeting macrophages: therapeutic approaches in cancer. Nat Rev Drug Discov. (2018) 17:887–904. doi: 10.1038/nrd.2018.169 30361552

[96] Yang H Zhang Q Xu M Wang L Chen X Feng Y. CCL2-CCR2 axis recruits tumor associated macrophages to induce immune evasion through PD-1 signaling in esophageal carcinogenesis. Mol Cancer. (2020) 19:41. doi: 10.1186/s12943-020-01165-x 32103760 PMC7045401

[97] Cui K Hu S Mei X Cheng M. Innate immune cells in the esophageal tumor microenvironment. Front Immunol. (2021) 12:654731. doi: 10.3389/fimmu.2021.654731 33995371 PMC8113860

[98] Mensurado S Blanco-Domínguez R Silva-Santos B. The emerging roles of γδ T cells in cancer immunotherapy. Nat Rev Clin Oncol. (2023) 20:178–91. doi: 10.1038/s41571-022-00722-1 36624304

[99] Hu Y Hu Q Li Y Lu L Xiang Z Yin Z. γδ T cells: origin and fate, subsets, diseases and immunotherapy. Signal Transduct Target Ther. (2023) 8:434. doi: 10.1038/s41392-023-01653-8 37989744 PMC10663641

[100] Patil RS Shah SU Shrikhande SV Goel M Dikshit RP Chiplunkar SV. IL17 producing γδT cells induce angiogenesis and are associated with poor survival in gallbladder cancer patients. Int J Cancer. (2016) 139:869–81. doi: 10.1002/ijc.v139.4 27062572

[101] Meraviglia S Lo Presti E Tosolini M La Mendola C Orlando V Todaro M. Distinctive features of tumor-infiltrating γδ T lymphocytes in human colorectal cancer. Oncoimmunology. (2017) 6:e1347742. doi: 10.1080/2162402X.2017.1347742 29123962 PMC5665062

[102] Wang J Lin C Li H Li R Wu Y Liu H. Tumor-infiltrating γδT cells predict prognosis and adjuvant chemotherapeutic benefit in patients with gastric cancer. Oncoimmunology. (2017) 6:e1353858. doi: 10.1080/2162402X.2017.1353858 29147601 PMC5674957

[103] Nguyen S Chevalier MF Benmerzoug S Cesson V Schneider AK Rodrigues-Dias SC. Vδ2 T cells are associated with favorable clinical outcomes in patients with bladder cancer and their tumor reactivity can be boosted by BCG and zoledronate treatments. J Immunother Cancer. (2022) 10(8). doi: 10.1136/jitc-2022-004880 PMC9413168 36002184

[104] Kumar AR Devan AR Nair B Vinod BS Nath LR. Harnessing the immune system against cancer: current immunotherapy approaches and therapeutic targets. Mol Biol Rep. (2021) 48:8075–95. doi: 10.1007/s11033-021-06752-9 PMC8605995 34671902

[105] Pérez-Ruiz E Melero I Kopecka J Sarmento-Ribeiro AB García-Aranda M De Las Rivas J. Cancer immunotherapy resistance based on immune checkpoints inhibitors: Targets, biomarkers, and remedies. Drug Resist Update. (2020) 53:100718. doi: 10.1016/j.drup.2020.100718 32736034

[106] Chan TA Wolchok JD Snyder A. Genetic basis for clinical response to CTLA-4 blockade in melanoma. N Engl J Med. (2015) 373:1984. doi: 10.1056/NEJMc1508163 26559592

[107] Rizvi NA Hellmann MD Snyder A Kvistborg P Makarov V Havel JJ. Cancer immunology. Mutational landscape determines sensitivity to PD-1 blockade in non-small cell lung cancer. Science. (2015) 348:124–8. doi: 10.1126/science.aaa1348 PMC4993154 25765070

[108] Chen C Wang C Li Y Jiang S Yu N Zhou G. Prognosis and chemotherapy drug sensitivity in liver hepatocellular carcinoma through a disulfidptosis-related lncRNA signature. Sci Rep. (2024) 14:7157. doi: 10.1038/s41598-024-57954-7 38531953 PMC10965927

[109] Song Z Cao X Wang X Li Y Zhang W Wang Y. A disulfidptosis-related lncRNA signature for predicting prognosis and evaluating the tumor immune microenvironment of lung adenocarcinoma. Sci Rep. (2024) 14:4621. doi: 10.1038/s41598-024-55201-7 38409243 PMC10897395

